# Ultrafast Laser Synthesis of Zeolites

**DOI:** 10.1002/adma.202415562

**Published:** 2025-04-17

**Authors:** Sezin Galioglu, Mehdi Hagverdiyev, Meryem M. Doğan, Özgün Yavuz, Ü. Seleme Nizam, Ghaith Makey, Aladin Şura, Mesut Laçin, Burcu Akata Kurç, Parviz Elahi, F. Ömer Ilday, Serim Ilday

**Affiliations:** ^1^ UNAM‐National Nanotechnology Research Center Bilkent University Ankara 06800 Türkiye; ^2^ Faculty of Electrical Engineering and Information Technology Ruhr University Bochum 44801 Bochum Germany; ^3^ Faculty of Physics and Astronomy Ruhr University Bochum 44801 Bochum Germany; ^4^ Micro and Nanotechnology Programme Middle East Technical University Ankara 06800 Türkiye; ^5^ Faculty of Engineering Özyeğin University İstanbul 34794 Türkiye

**Keywords:** multiphoton absorption, nonlinear light–matter interactions, nucleation and growth, self‐assembly, spatiotemporal control, ultrafast laser synthesis, zeolite

## Abstract

Research demonstrates that zeolite nucleation and growth can be controlled by fine‐tuning chemical composition, temperature, and pressure, resulting in structures with diverse porosities and functionalities. Nevertheless, current energy delivery methods lack the finesse required to operate on the femto‐ and picosecond timescales of silica polymerization and depolymerization, limiting their ability to direct synthesis with high precision. To overcome this limitation, an ultrafast laser synthesis technique is introduced, capable of delivering energy at these timescales with unprecedented spatiotemporal precision. Unlike conventional or emerging approaches, this method bypasses the need for specific temperature and pressure settings, as nucleation and growth are governed by dynamic phenomena arising from nonlinear light–matter interactions, such as convective flows, cavitation bubbles, plasma formation, and shock waves. These processes can be initiated, paused, and resumed within fractions of a second, effectively “freezing” structures at any stage of self‐assembly. Using this approach, the entire nucleation and growth pathway of laser‐synthesized TPA‐silicate‐1 zeolites is traced, from early oligomer formation to fully developed crystals. The unprecedented spatiotemporal control of this technique unlocks new avenues for manipulating reaction pathways and exploring the vast configurational space of zeolites.

## Graphical abstract

The study presents a novel ultrafast laser‐driven synthesis technique for zeolites directly in liquid. Laser pulses create a microscopic reactor that controls reaction kinetics at femto‐ and picosecond timescales. Nonlinear light‐matter interactions drive nucleation and growth, allowing the process to be paused at any stage of self‐assembly.

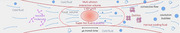

## 1 Introduction

Zeolites are crystalline inorganic materials with well‐dispersed and systematically arranged nano and/or micron‐sized pores. Their chemical composition, pore dimensions, and high surface area can be tailored to yield diverse functionalities such as selective adsorption and separation, carbon dioxide capture, host‐guest assembly, catalysis, mass transport facilitation, ion exchange, and acting as softeners and gas sensors, which are important in various scientific and industrial applications.^[^ ^1^, ^2^, ^3^ ^]^

Around 40 zeolitic frameworks are found in nature. Millions of different zeolite frameworks have been identified computationally, but only 256 were synthesized in the laboratory, as recognized by the International Zeolite Association (IZA).^[^ ^4^ ^]^ Although new zeolitic frameworks are synthesized regularly, repeatability, stability, and optimization have been reported as major challenges because the final framework structure and functionalities are sensitive to minor variations in the synthesis parameters.^[^ ^4^, ^5^, ^6^, ^7^ ^]^ We argue that these problems are related to the energy delivery mechanism and how it drives and controls nucleation and growth.

Heat diffuses gradually into the precursor solution in hydrothermal synthesis processes. Nonuniform temperatures and reactant concentrations in liquid volume spatially alter the nucleation and growth conditions as more or less favorable. This hinders uniform particle formation and produces a relatively broad crystal size distribution.^[^ ^8^, ^9^ ^]^ Conversely, in microwave synthesis, energy is delivered directly to the reactants, resulting in localized heat points throughout the liquid volume. However, lacking an efficient mechanism to dissipate the excess heat, the process creates an unstable, locally superheated liquid, highly sensitive to even minor perturbations, forming low‐quality zeolite crystals.^[^ ^9^, ^10^ ^]^ Alternatives to these conventional synthesis techniques, such as gamma‐ray,^[^ ^11^ ^]^ high‐energy electron beam irradiation,^[^ ^12^ ^]^ and ultaviolet radiation,^[^ ^13^ ^]^ use high‐energy radiation to induce ionization to form hydroxyl free radicals, lowering activation energies and accelerating nucleation and growth. However, complex processes, safety concerns, repeatability issues, and other limitations have hindered their widespread adoption.^[^ ^11^, ^12^ ^]^

The ultrafast laser synthesis approach introduces an entirely different energy delivery mechanism, rendering absolute temperature and pressure values less critical. A tiny ultrafast reactor at the focus of a femtosecond laser beam can process a liquid volume of a few thousand µm^3^ with extremely high spatiotemporal energy inputs and thermal gradients building up to 10⁶ K mm^−1^ within several 100 fs, aligning with the timescales of silica polymerization and depolymerization reactions. Nonlinear light absorption (multiphoton) by the precursor solution generates a cascade of events that occur in rapid succession.^[^ ^14^, ^15^ ^]^ These include plasma generation, cavitation bubble formation, the emergence of high‐speed convective flows,^[^ ^16^, ^17^ ^]^ and shock (pressure) waves,^[^ ^18^, ^19^ ^]^ which facilitate controlled nucleation and growth of nearly uniform zeolite crystals. Using this technique, various zeolitic frameworks are synthesized, including Mobil type five (MFI), Linde type A (LTA), and Faujasite type (FAU), with different pore sizes and configurations and crystallinities surpassing 90% with a wt.% yield of ≈70%.

Uniquely, the synthesis can be stopped within fractions of a second by turning off the laser. The laser is electronically controlled and can be turned on and off within microseconds. This can be repeated as much as needed, allowing for iterative sample collection. Turning off the laser rapidly terminates all the laser‐induced dynamic phenomena, halting the nucleation and growth. Since no significant residual heat remains in the system to assist further chemical reactions, the structures formed up to that point are effectively “frozen” in their self‐assembled state. Samples can easily be collected for diagnostic analysis, after which synthesis can be resumed by turning on the laser. Using this capacity, the sequential evolution of the synthesis pathway of TPA‐silicate‐1 zeolites is followed from the formation of early oligomers to the emergence of fully grown crystals.

## 2 Results and Discussion

### 2.1 The Energy Delivery Mechanism

**Figure 1:**
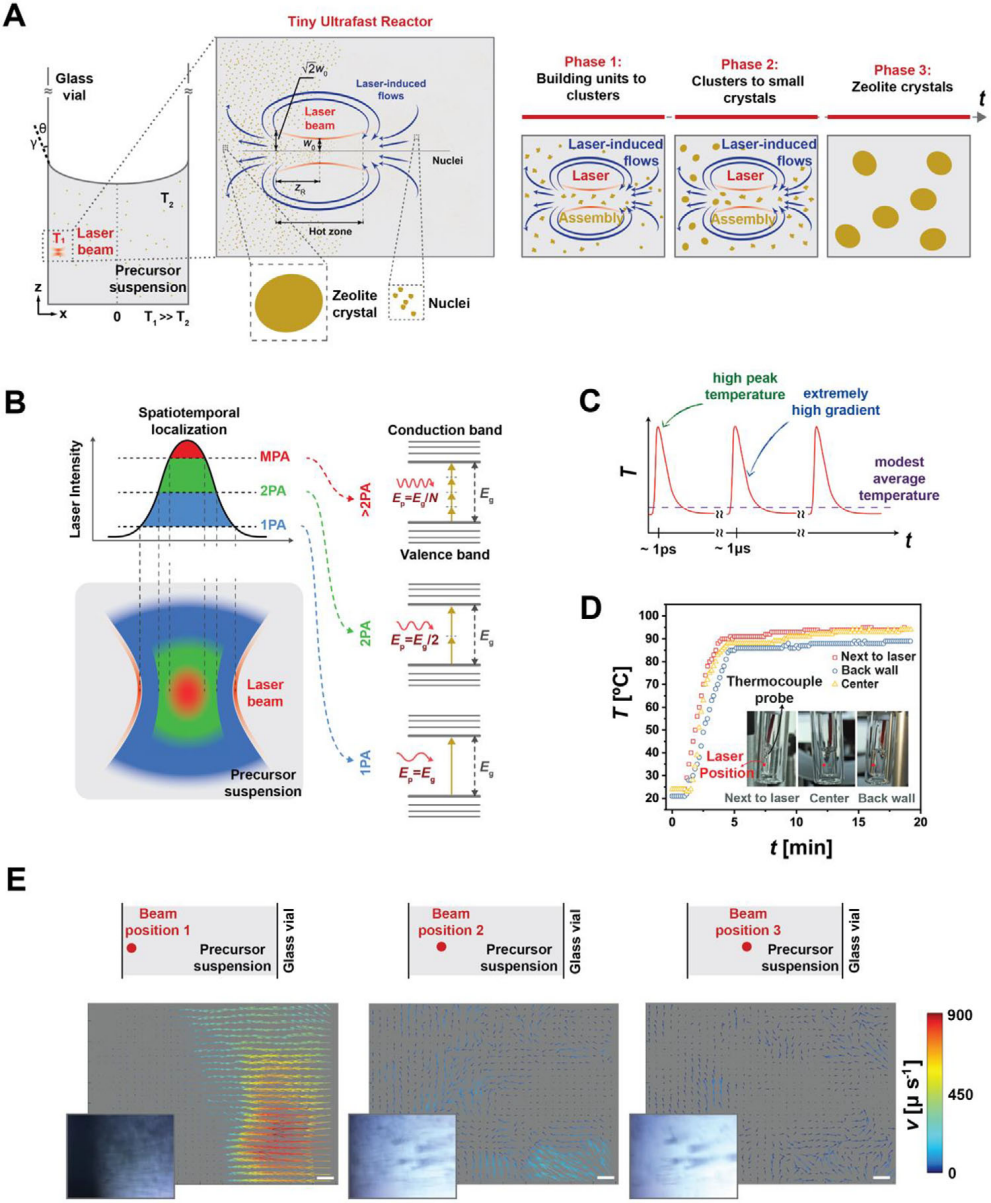
A) Schematics illustrating the working mechanism of a tiny ultrafast reactor at the glass–liquid interface. Reactions are initiated in the hot zone near the beam waist (ω_0_), which is twice the Rayleigh length (*z*_R_), the distance along the beam's propagation direction from the waist to the position where the cross‐section area is doubled. The zeolitic crystals are produced in this tiny reactor and distributed to the full liquid volume via laser‐induced convective flows, which emerge due to extreme thermal gradients and surface tensions (*γ*) B) A simplified sketch depicts linear and nonlinear absorption processes. 1PA, 2PA, and MPA indicate the absorption of one, two, and multiple photons. 1PA (linear absorption) creates an hourglass‐shaped absorption profile (blue) in the precursor solution, while MPA (nonlinear absorption) is extremely localized (red). C) A cartoon‐like illustration of a temperature profile induced by an ultrafast pulse train. Each pulse delivers identical energy at precise intervals. Upon each pulse's arrival, the local temperature spikes almost instantaneously (within femtoseconds), creating extreme temporal thermal gradients. This temperature increase lasts only as long as the pulse, after which it begins to cool rapidly until the next pulse arrives a few microseconds later. D) Recorded temperature readings with the thermocouple positioned at the front wall near the beam (position 1), the center of the bulk liquid (position 2), and the back wall away from the beam (position 3) showing that time‐averaged local temperature remains steady ≈95 °C. E) Average laser‐induced flow fields were calculated using particle image velocimetry (PIV) analysis of video recordings, where the laser beam was focused at three positions: at the glass–liquid interface (beam position 1), near the interface (beam position 2), and within the bulk liquid (beam position 3). The insets show frames captured from the video recordings. The velocity color bar shows the lower bound for the fluid velocity calculations.

The tiny ultrafast reactor is created by focusing a femtosecond laser beam at the glass–liquid interface (**Figure** **1**A). This approach mirrors our previous work on quasi‐2D colloidal self‐assembly^[^ ^16^, ^17^ ^]^ where laser pulses are nonlinearly absorbed through multiphoton absorption, generating a cascade of events that drive the self‐assembly of a variety of active and passive colloidal particles and living organisms.

Multiphoton absorption occurs when multiple photons are absorbed simultaneously by the medium, enabling an electron to transition to a higher energy state, even if the energy of each individual photon is insufficient to bridge the gap between energy levels (Figure 1B). This process requires high‐intensity light, such as our tightly focused ultrafast laser beam, where the extremely high photon density at the focal point allows multiple photons to interact with a single atom or molecule on the timescale of its electronic response. This confines energy deposition to a significantly smaller volume (≈10^3^ µm^3^) than the focal volume (>10^3^ µm^3^). As a result, the spatial temperature gradients exceed 10⁶ K mm^−1^ within the tiny ultrafast reactor but only for a fleeting moment (in the order of the pulse duration) (see Supplementary Information). This extreme localization creates a steep spatiotemporal thermal gradient (Figure 1C). As a result, the time‐averaged local temperature remains steady around a much lower value (Figure 1D).

Spatiotemporal temperature gradients, together with surface tensions at the glass–liquid interface (Figure 1A), drive and indefinitely sustain high‐speed Marangoni‐type convective flows (Figure 1E) along the beam propagation axis as long as the laser beam is incident. These flows can be clearly observed in real‐time experimental recordings (Videos S1 and S2, Supporting Information). A simple calculation (see Supporting Information) shows that the entire chemical reaction occurs on a microsecond timescale. We assume that the local temperature remains substantially above the average temperature of 95 °C only within the interaction volume and that the precursor solution is mixed well enough during the experiment. The total number of pulses during a typical experiment of 240 min and at 200 kHz repetition rate is 3 billion. The ratio of the interaction volume to the total solution is similarly ≈0.4 billion. Hence, any given portion of the precursor solution is processed at high temperatures for the timespan of ≈10 pulses, or ≈50 µs.

The working dynamics of this unique energy delivery mechanism in self‐assembling zeolitic crystals are discussed in the next section. However, before proceeding, we would like to emphasize the unique role of ultrafast lasers in energy delivery.

**Figure 2:**
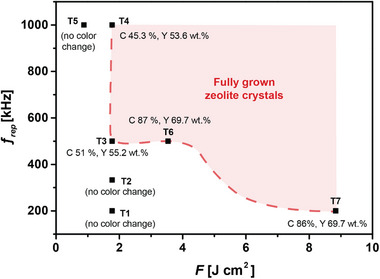
Graph depicting the optimal laser parameters for the formation of fully‐grown zeolite crystals. The repetition rate (*f_rep_*) increases while maintaining the total deposited energy and pulse fluence (*F*) constant (T1, T2, T3, T4). *F* is halved while keeping the total deposited energy and *f_rep_* unchanged (T5). *F* is doubled while maintaining the total deposited energy and *f_rep_* constant (T6). *F* is significantly increased while keeping *f_rep_* constant (T7). The crystallinity (C) and weight percentage yield (Y) values are provided for comparison in each trial.

Historically, significant progress has been achieved in the ultrafast synthesis of colloidal nanoparticles in liquid,^[^ ^20^ ^]^ where the nanoparticles are ablated from a solid target immersed in the liquid. Subsequent hydrothermal synthesis is typically used following the laser treatment to grow the nuclei into mature crystals.^[^ ^21^ ^]^ The potential of ultrafast lasers in synthesizing zeolites (or other inorganic nanomaterials) by driving chemical reactions directly and fully in liquid has not been thoroughly explored. We believe that the attempts to process the entire precursor solution in a single step likely faced two major challenges: 1) Insufficient energy deposition to the solution, a challenge compounded by the lack of detailed parameters in existing literature, such as pulse fluence and repetition rate (by contrast, see our optimization analysis in **Figure** **2**; Table S1, Supporting Information). 2) The difficulty in initiating nonlinear light–matter interactions necessary for processing the entire liquid volume. This issue, while not always explicitly stated, is suggested by experimental setup diagrams, where the beam is often pointed (which might not be tightly focused) arbitrarily within the bulk liquid, which is suboptimal for efficient multiphoton absorption by the solution.

We conducted further experiments to support arguments on the second challenge. A typical synthesis process captured in Videos S1 and S2 (Supporting Information) visualizes the flow fields at three distinct beam positions: at the glass–liquid interface (left), in close proximity to the interface (middle), and closer to the center of the bulk liquid (right). An average speed is computed over a smaller time frame to highlight the distinct characteristics of the flow fields for the three beam positions, which appear at the end of Video S2 (Supporting Information) and are shown in Figure 1E. Significantly, high‐speed flows manifest exclusively when the beam is focused at the glass–liquid interface, whereas seemingly unstructured slow flows are observed away from the interface. This is expected as surface tensions (Figure 1A) promote the creation of Marangoni flows at the interface,^[^ ^16^, ^17^ ^]^ a condition absent at the two beam positions away from the glass walls. Moreover, the transition of the precursor suspension color from transparent to opaque white in Video S1 (Supporting Information) signifies the saturation of the liquid volume with fully‐grown crystals, which is exclusively achieved when the beam is focused at the interface under the same reaction time.

Mechanical stirring of the precursor solution in conventional hydrothermal synthesis has been studied by gradually rotating the autoclave, typically within a speed range of 10–20 rpm.^[^ ^22^, ^23^ ^]^ This approach has yielded positive outcomes, such as slightly accelerated crystal growth and a relatively narrow size distribution. We further asked if the benefits reported here could be solely attributed to the laser‐induced convective flows. To examine this, the laser beam is deliberately directed onto the bulk liquid (avoiding the glass–liquid interface) to prevent the formation of Marangoni flows along with all the dynamic phenomena resulting from multiphoton absorption of pulses. Instead, the solution is mechanically mixed using a magnetic stirrer (Video S3, Supporting Information). As anticipated, no noticeable change in color was observed within the precursor solution, even after tripling the laser exposure time compared to the typical laser synthesis conditions reported here.

As for using other lasers for syntheses, such as continuous‐wave (CW) and nanosecond, we argue that the attempts would likely fail, as these lasers cannot replicate the dynamic conditions (e.g., flows, bubbles, etc.) that femtosecond lasers achieve. CW lasers function more like inefficient furnaces, gradually heating the precursor solution, which only slowly promotes nucleation and growth. Although nanosecond lasers can induce multiphoton absorption, they require significantly higher pulse fluences due to their longer pulse durations and lower peak intensities. This extended pulse duration causes thermal diffusion, leading to heat buildup in the material, which can trigger undesirable thermal effects, even melting or ablating the glass vial. This compromises precision, especially in applications requiring spatiotemporal control.

### 2.2 The Self‐Assembly Mechanism

The initial phase involves initiating silica polymerization‐depolymerization reactions within the hot zone of the laser beam (Figure 1A), effectively serving as a tiny ultrafast reactor for zeolite synthesis. The local temperature in the reactor is periodically elevated, momentarily reaching extremely high levels that significantly accelerate the chemical reaction.

Early‐stage oligomer rings and condensed units, produced with each laser pulse, are systematically dispersed throughout the precursor solution via convective flows. The reactor volume is on the order of several thousand µm^3^, less than a billionth of the total liquid volume. As a result, any self‐assembled unit reaches thermal equilibrium with the bulk long before it re‐enters the reactor. This exceptionally small reactor‐to‐bulk volume ratio ensures that, while local temperatures within the reactor are extremely high, the average temperature across the entire system remains low, around the measured value of 95 °C (Figure 1D).

The energy input by the femtosecond laser pulses is not only used in chemical synthesis but also in generating and sustaining laser‐induced dynamic phenomena. A rapid buildup of free electrons upon multiphoton absorption triggers optical breakdown. This results in the formation of a transient plasma at extremely high temperatures and pressures. Plasma increases the concentration of reactive species and further promotes nucleation and growth. As the plasma expands, it displaces the surrounding liquid, generating cavitation bubbles. Together with laser‐induced convective flows, these buoyant bubbles enhance the efficient mixing of the precursor solution, promoting faster and nearly uniform crystal growth. The bubbles grow due to the extreme pressure difference. Some subsequently collapse, emitting shock waves that further influence the surrounding medium. Extreme pressures propagate, further assisting nucleation and growth. This sequence of events plays a critical role in energy transfer, fluid dynamics, and chemical reactions within the tiny ultrafast reactor.

As the reactor is the only hot zone and the bulk liquid is significantly colder, convective flows continually move between the reactor and the bulk liquid. This is crucial in continually transporting previously distributed nuclei and particles back to the reactor, where they grow further during each iteration, evolving into larger structures and maturing into nearly mono‐sized and discrete zeolite crystals (see Supporting Information). This unparalleled efficiency sets ultrafast laser synthesis apart from other methods in the field.

Another great benefit of ultrafast laser synthesis in controlling self‐assembly stems from the dynamic control over the rate of energy delivery—a capability absent in both conventional (such as hydrothermal and microwave methods) and recently developed approaches (including assembly‐disassembly‐organization‐reassembly,^[^ ^24^ ^]^ seed‐directed secondary growth,^[^ ^25^ ^]^ ultraviolet,^[^ ^13^ ^]^ gamma‐ray,^[^ ^11^ ^]^ and high‐energy electron beam irradiation.^[^ ^12^ ^]^ This is achievable by adjusting pertinent laser parameters such as average laser power, peak power, and pulse energy fluence.

Furthermore, the process can be halted and resumed by turning the laser on and off (Video S4, Supporting Information) for rapid cooling and heating of the liquid medium, exerting a profound influence on the kinetics of the chemical reaction. Rapid cooling is possible because the laser energy is delivered only to the focal volume, and excess heat is immediately carried out via convective flows and distributed to the bulk liquid. If the laser beam is turned off, no significant heat source remains to support further nucleation and growth (see Supporting Information). That way, the self‐assembled structures up until that point are effectively frozen and preserved. Reintroducing the laser pulses rapidly provides the energy to promote the chemical reactions and resume the self‐assembly process.

### 2.3 Characteristics of Ultrafast Laser Synthesized Zeolites

**Figure 3:**
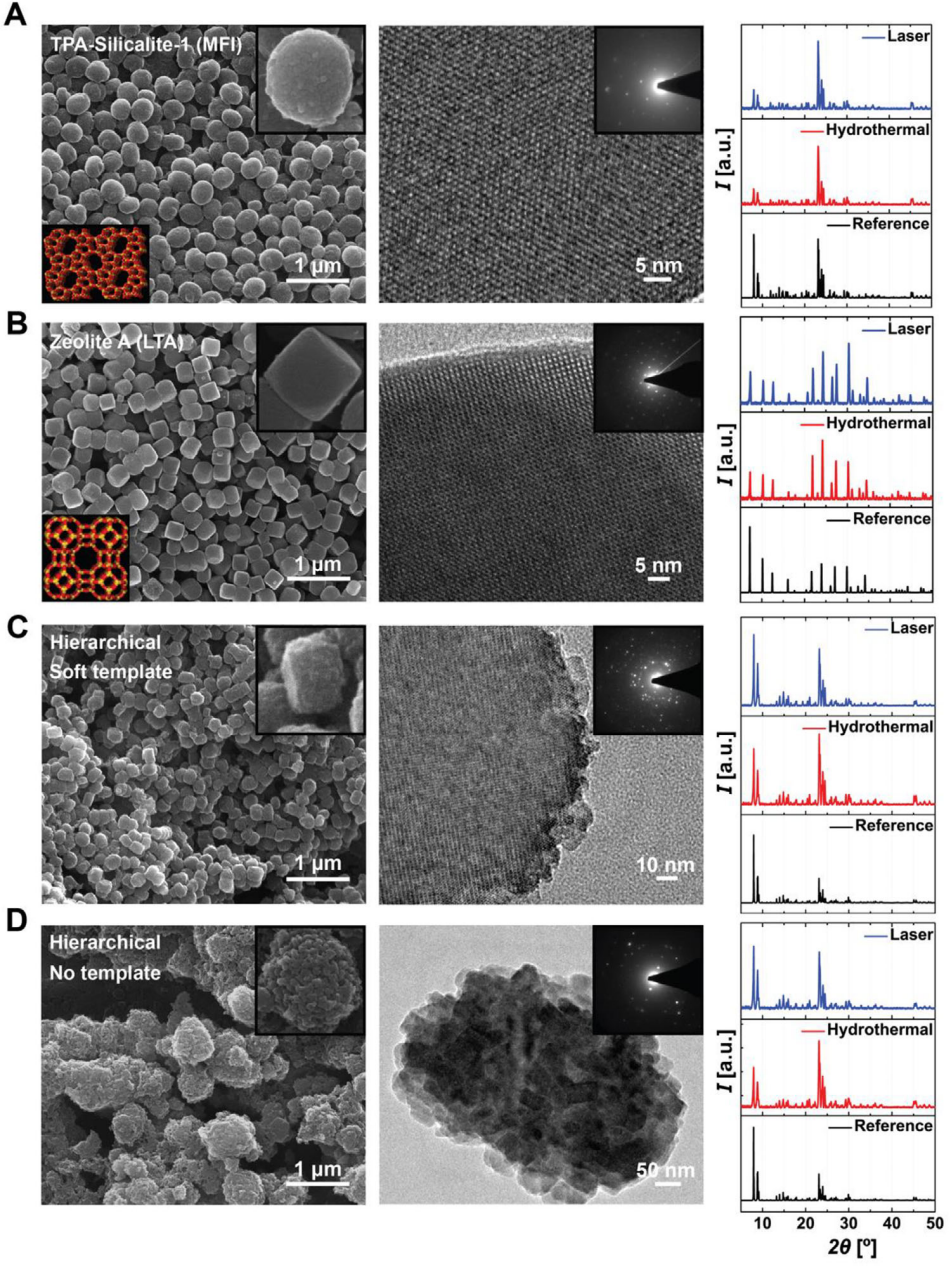
SEM (left) and TEM (middle) images of A) TPA‐Silicalite‐1 (MFI type), B) Zeolite A (LTA type), C) hierarchical ZSM‐5 with a soft template, and D) mesoporogen‐free hierarchical ZSM‐5 zeolites. The left panel insets show unit cell structures (lower left) and SEM images of individual crystals (upper right). The middle panel insets show SAD patterns. XRD spectrums of the laser‐ (blue, upper) and hydrothermal‐synthesized (red, middle) zeolites are compared to their International Zeolite Association (IZA) references (black, lower layer) at the rightmost panel. TPA‐Silicalite‐1 crystals are obtained after 180 min reaction time.

A selection of laser‐synthesized zeolites is shown in **Figure** **3**, with their crystalline identities confirmed via XRD spectroscopy (see also Figures S3 and S4, Supporting Information for MFI‐type Zeolite Socony Mobil‐5 (ZSM‐5) and for FAU‐type Zeolite‐Y). To ensure the reliability and reproducibility of the synthesized zeolites, we conducted over 120 experiments, consistently yielding similar results (see Figures S5–S7, Supporting Information for the results of representative experiments).

Our focus centered on TPA‐silicalite‐1, a well‐documented and extensively studied model zeolite. To draw quantitative comparisons, half of the prepared precursor solution was subjected to laser synthesis for 3 h, while the other half underwent a typical hydrothermal synthesis for 48 h. Comparative analysis of the average crystal size distribution between laser‐ and hydrothermal‐synthesized zeolites reveals distinct outcomes.

The relatively large crystal sizes and narrow size distribution of ultrafast laser‐synthesized zeolites result from the unique energy delivery mechanism discussed in the previous two sections. Heat and the reactants diffuse and transport significantly more slowly in conventional hydrothermal synthesis, creating uneven nucleation and growth conditions resulting in larger size variations.

Laser synthesis yields a narrow distribution with larger crystal sizes (Figure S8A,C, Supporting Information). In contrast, hydrothermal synthesis results in a broader distribution featuring relatively smaller ones (Figure S8B,D, Supporting Information). Notably, akin to hydrothermal synthesis, reducing the average crystal size in laser synthesis is feasible by modifying the molar formula's water content from M1 (Figure S8, Supporting Information) to M2 (Figure S11, Supporting Information) and M3 (Figure S10, Supporting Information) (see also Figure S11 and Table S2, Supporting Information).

Thermal stabilities, as measured through thermogravimetric (TGA) and differential thermal (DTA) analyses for both laser and hydrothermally synthesized (Figure S12, Supporting Information) zeolites, yield comparable outcomes with water removal of 4.2% at ≈109 °C in the case of laser and 3.4% at ≈76.4 °C in hydrothermal synthesis and removal of 12.1% TPA content for both methods at ≈334 °C in the case of laser and 329 °C in hydrothermal synthesis.

Crystallinity and product yield calculations suggest similar results for laser‐ and hydrothermally synthesized zeolites (Table S2, Supporting Information). N_2_ adsorption‐desorption curves (Figure S13, Supporting Information) manifest a Type I isotherm, indicative of a microporous structure (pore size <2 nm)^[^ ^26^ ^]^ with the Brunauer–Emmett–Teller (BET) surface area of 335.5 m^2^ g^−1^ (falling within the typical range of 332–400 m^2^ g^−1^)^[^ ^27^, ^28^, ^29^, ^30^ ^]^ for laser synthesized TPA‐silicalite‐1 crystals. In accordance with IZA reported values, DFT analyses show that the pore volume and effective pore radius of the laser‐synthesized TPA‐silicalite‐1 crystals are 0.164 cm^3^ g^−1^ and 2.88 Å, respectively (Figure S14, Supporting Information).

### 2.4 Nucleation and Growth Pathways

**Figure 4:**
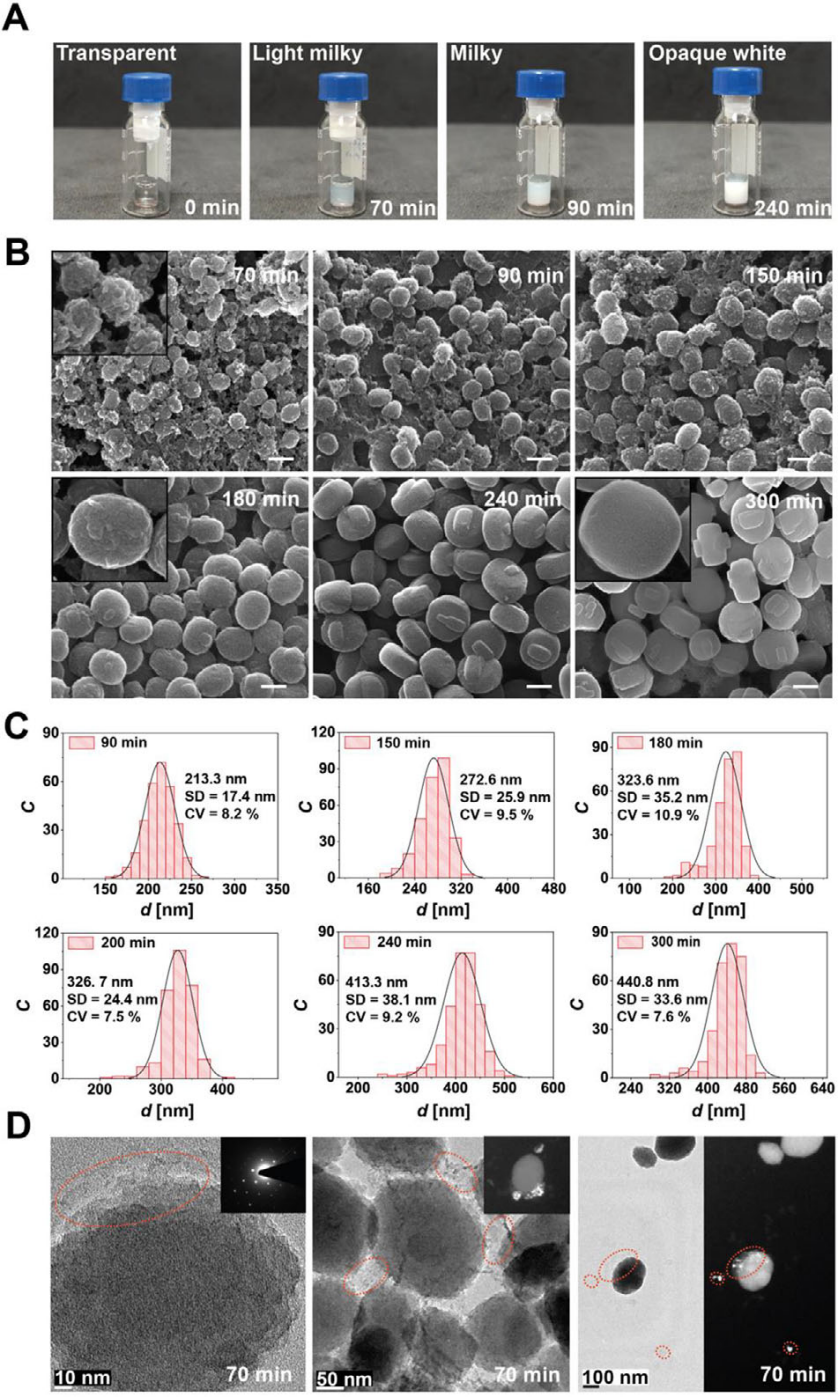
A) Photographs taken at different stages of laser synthesis show color change from transparent to milky white and opaque white as the reaction time increases. B) SEM images show the evolution of crystals from rough to smoother surfaces as the reaction time increases. C) Average crystal size distribution plots for zeolites synthesized in various reaction times. *C* denotes particle counts. D) TEM images of crystals were obtained after 70^th^ min reaction time shows rough surface contours (dashed red ellipses). The phase contrast TEM images show that these marked regions appear bright, confirming their crystalline nature.

We sampled the process at intervals from the 20^th^ to the 300^th^ min by turning the laser off and on and identified two distinct regimes for nucleation and growth. Throughout the synthesis, no observable color change occurred in the precursor solution until the 70^th^ min. The initially light milky color at 70^th^ min transformed to a milky hue by the 90^th^ min, later evolving into an opaque white appearance by the 240^th^ min (**Figure** **4**A).

Subsequent SEM images (Figure 4B) and average crystal size distributions (Figure 4C) reveal where a high‐degree network of smaller crystals with rough surfaces gradually transitioned into larger, discrete crystals with smoother surfaces. SEM and TEM images of samples collected at the 70^th^ min suggest that individual crystals are formed by aggregating smaller crystals, each with an average size of ≈15 nm. Since those on the surface and contributing to the network by bridging crystal aggregates are electron‐beam sensitive, phase‐contrast TEM images were performed, revealing a bright appearance and confirming their crystalline nature (Figure 4D).

**Figure 5:**
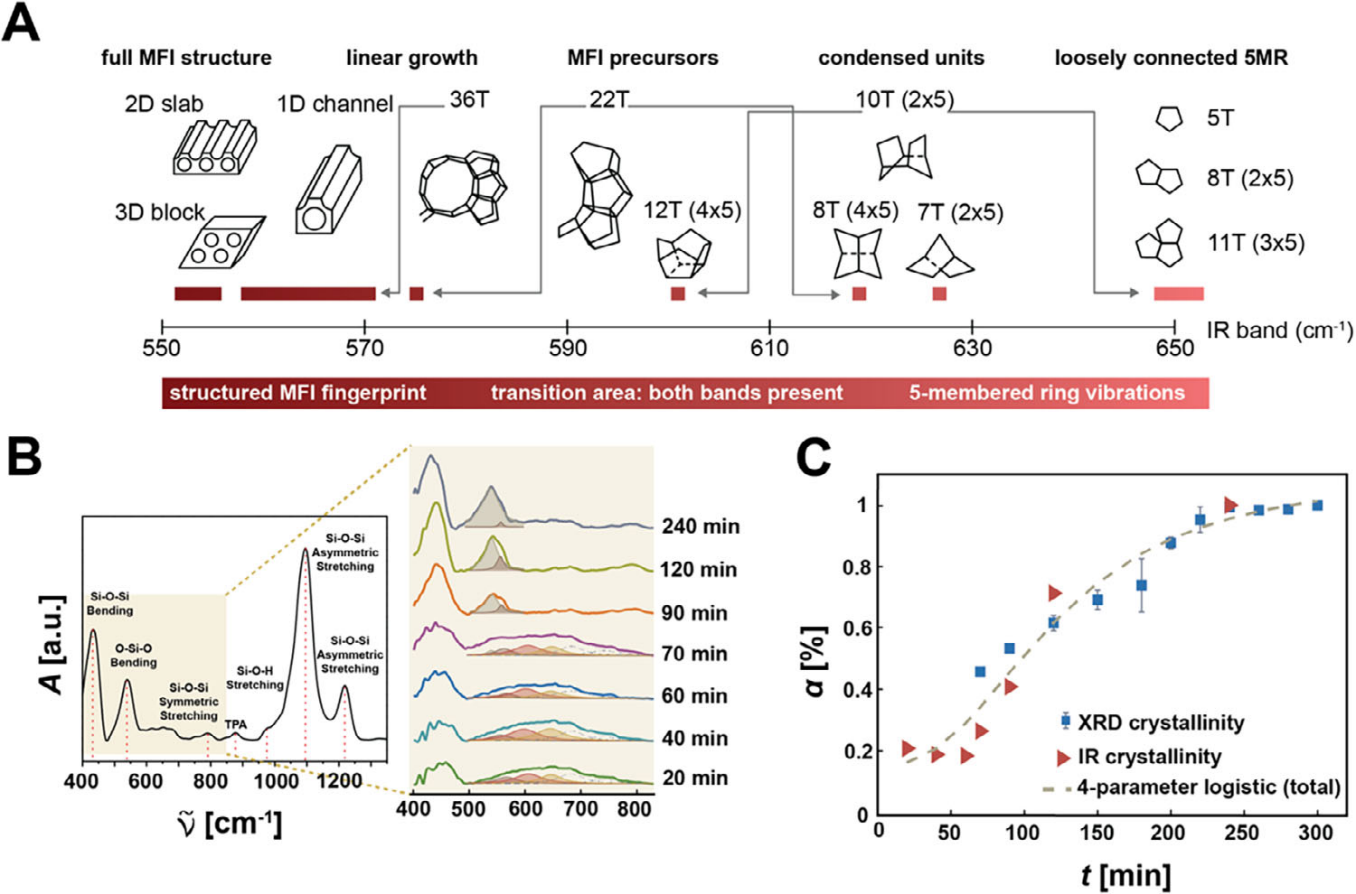
A) Molecular dynamics simulations show the IR band shift corresponding to different nucleation and growth stages of TPA‐Silicalite‐1 zeolites. Reproduced with permission.^[^ ^28^ ^]^ 2008, American Chemical Society. B) The IR absorbance spectrum of zeolites with signal contributions from loosely connected 5‐membered ring structures (yellow‐filled peak centered at wavenumber ≈650 cm^−1^) to condensed units of 10T and 22T structures (red‐filled peak centered at ≈600 cm^−1^), 36T MFI precursors (brown‐filled peak centered at ≈560 cm^−1^), and fully grown MFI structures (grey‐filled peak centered at ≈540 cm^−1^). C) The graph shows the sigmoidal growth of the crystallinity index (*α*) calculated from ATR‐FTIR (red triangles) and XRD (blue squares) to synthesis time.

Moreover, to elucidate the chemical transformations occurring during the formation of these minute crystals, we scrutinized the Si‐O‐Si and O‐Si‐O bending and the Si‐O‐Si symmetric stretching signals using ATR‐FTIR spectroscopy. Comparative analysis with molecular dynamics simulations^[^ ^28^, ^29^, ^30^ ^]^ (**Figure** **5**A,B) reveals the presence of loosely connected 5‐membered ring structures (yellow‐filled peak), condensed units of 10T and 22T structures (red‐filled peak), and 36T MFI precursors (brown‐filled peak) as early as 20^th^ min of the synthesis. Fully grown MFI structures appear at the 60^th^ min (grey‐filled peak) (see also Figure S15, Supporting Information). The evolution of peak relative intensities is tracked until the 300^th^ min of the reaction. Initially, the signal from early ring vibrations diminishes until the 70^th^ min and then ceases at the 90^th^ min, marking the complete consumption of small oligomers. Concurrently, the signal contribution from MFI precursors increases as they assimilate the early oligomers for their self‐assembly. After the 70^th^ min, the signal from fully grown MFI structures increases drastically by consuming the MFI precursors. Accordingly, the signal contribution of the MFI precursors diminished significantly.

We calculated the crystallinity of liquid samples collected at 20 < *t* < 300 min of the synthesis from XRD data and also from ATR‐FTIR analyses (Figure 5C) to correlate them with the self‐assembled structures calculated through the molecular dynamics simulations (Figure 5B). Our crystallinity analyses revealed fast and slow sigmoidal growth dynamics for MFI precursor structures and fully‐grown crystals, respectively.

The consumption of early oligomers by the growing MFI precursor structures until the 90^th^ min was much faster than that of MFI precursors by fully‐grown zeolite crystals. This is so because strong nonlinear light–matter interactions that drive the nucleation and growth are modified when the precursor solution transitions from transparent to opaque white, affecting the growth rate. Multiphoton absorption is the dominant absorption mechanism when the medium is transparent, and we start our experiments with a transparent precursor solution rather than its gel‐like form, typically used in conventional hydrothermal synthesis.

The initial transparent solution turns milky white at ≈90 min (Figure 4A), corresponding to where the initial fast growth rate slows in the crystallinity graph in Figure 5C. The slower growth rate eventually plateaus ≈240 min when the precursor solution is opaque white, indicating strong scattering.

### 2.5 Versatility of the Ultrafast Laser Synthesis Technique

**Figure 6:**
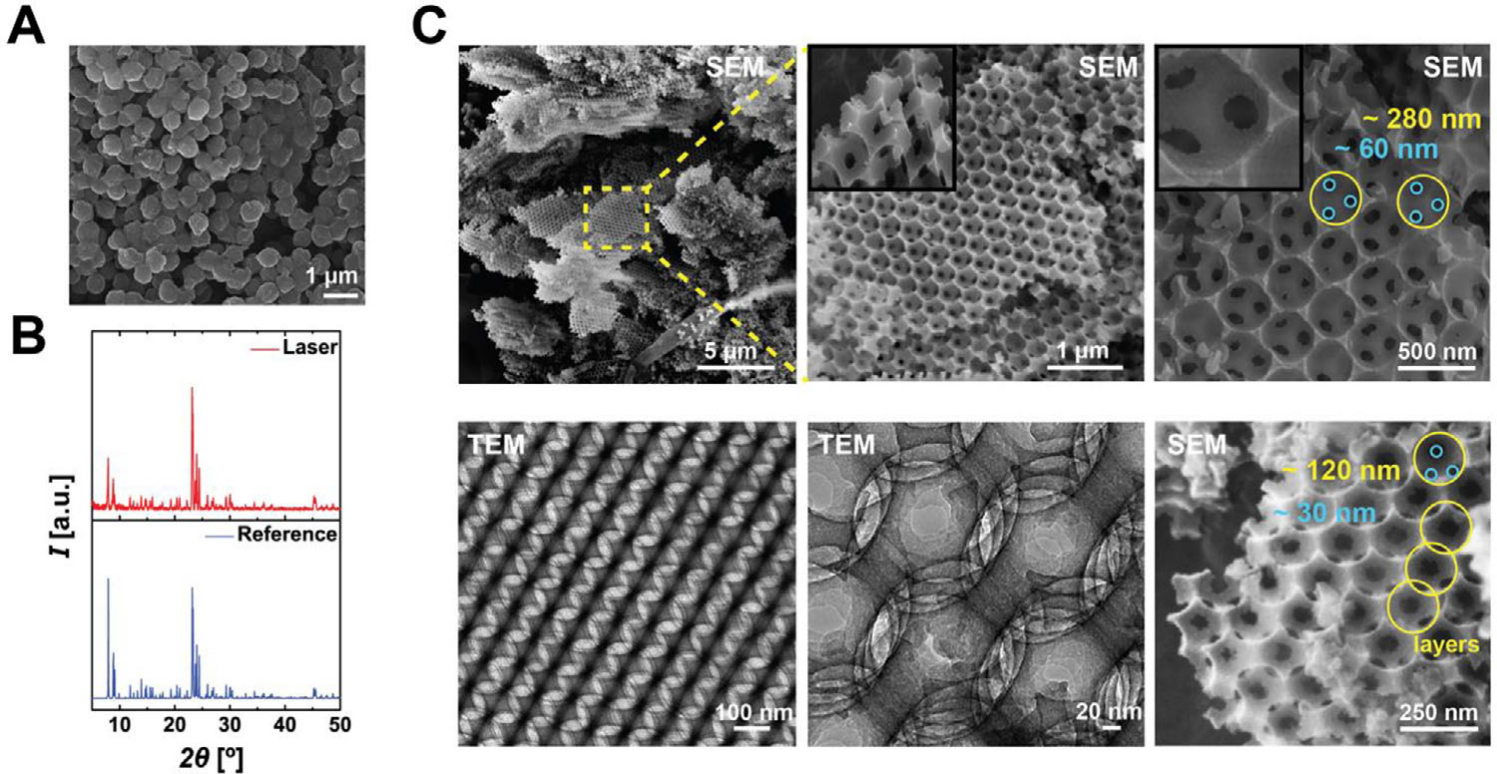
A) SEM image and B) XRD pattern showing laser‐synthesized TPA‐Silicalite‐1 without the preliminary aging step. C) SEM and TEM images showcase laser‐synthesized hierarchical structures.

Since femtosecond laser pulses provide energy within the relevant timescales of zeolite polymerization‐depolymerization reactions, it becomes feasible to synthesize zeolites without the customary aging step. Typically, in zeolite synthesis, the precursor solution undergoes a standard practice of being kept at room temperature for 24 h post‐chemical mixing. To show that this step might not be necessary, we applied the laser immediately after chemical mixing and successfully synthesized TPA‐Silicalite‐1 crystals (**Figure** **6**A,B).

Another distinctive feature of utilizing ultrafast lasers is the potential to leverage collective pulse‐matter interactions for synthesizing diverse topologies and functionalities, thereby further exploring the configurational phase space of zeolites. This is a new interaction regime with different kinetics whereby multiple pulses simultaneously interact with the matter instead of a single pulse. To experiment with this idea, we utilized a home‐built ultrafast burst‐mode laser.^[^ ^31^ ^]^ Figure 6C shows that our initial findings with this new light–matter interaction regime produced hierarchical structures with pore openings spanning ≈30 to 60 nm and 120 to 280 nm. The large field‐of‐view SEM images reveal an abundance of layer‐by‐layer formation of these structures, while higher‐magnification images highlight their hierarchical porous architecture. The pore structure and periodicity of the hierarchy are further evident in the TEM images.

## 3 Conclusion

The ultrafast laser synthesis technique reported here is a potential paradigm shift in zeolite synthesis. Femtosecond laser pulses provide extremely localized energy in space and time, allowing high‐precision control over energy delivery, reaction kinetics, and chemical synthesis. Energy is delivered at timescales compatible with the natural timescales of zeolite polymerization and depolymerization reactions, which makes it possible to even eliminate the time‐consuming aging step. High spatiotemporal precision prevents unwanted heat accumulation and thermal diffusion, promoting ultrafast synthesis of nearly uniform, high‐quality, high‐yield crystals. The technique opens new possibilities for producing diverse zeolitic structures and topologies. Various zeolite configurations may be explored by adjusting laser parameters without altering the chemical composition. Additionally, collective light–matter interactions such as the one facilitated by burst‐mode lasers can be employed to explore more complex topologies that may be difficult or impossible to achieve using conventional methods, such as millions of computer‐generated zeolitic frameworks. We postulate that the technique introduced here is not limited to zeolite synthesis as laser‐induced dynamic phenomena driving the chemical synthesis are not specific to the chemicals used in the process. Synthesis of a broad range of inorganic materials should be possible.

## 4 Experimental Section

### Experimental Setup

The precursor solution was housed in a glass insert (Supelco, Merck, 0.75 mL, 7 mm in diameter), which was encased within a chromatography glass vial (IsoLab, 4 mL, N13, 14.8 mm diameter) securely positioned using an adjustable sample holder attached to its cap (Video S1, Supporting Information). An ultrafast laser beam (Spectra‐Physics, Spirit One, 1040–8–SHG) was focused at the glass–liquid interface. The laser operates at a central wavelength of 1040 nm, with a pulse duration of 300 fs and a repetition rate of 200 kHz. The beam size was measured to be 9 µm using a beam profiler (Thorlabs BP209‐IR2/M). To generate Figure 2, the total deposited energy or the pulse fluence was fixed to investigate the impact of laser parameters on zeolite formation. Consequently, the average laser power was varied from 2.5 to 5 W and adjusted the repetition rate between 200 kHz and 1 MHz for these experiments.

### Video Recordings and Analyses

The experiments were captured using CMOS scientific (DCC1645C‐HQ, Thorlabs) and Canon DSLR (Eos) cameras, employing a 10× Nikon objective for imaging. A white light source was positioned on the optical table at an appropriate angle to illuminate the glass vial and enhance visibility. Particle Image Velocimetry (PIV) analyses were conducted on recordings with a frame rate of ≈30 fps and a resolution of 1116 × 891 pixels, utilizing DynamicStudio Software from Dantec Dynamics over ≈420 frames. Grayscale images of velocity fields were generated for adaptive PIV analyses to calculate the 2D velocity fields on a 2D grid with tiles sized 16 × 16 pixels. The average displacements in the *x*‐ and *y*‐directions were then extracted using the software's MATLAB link option and converted to velocity vectors in units of µm s^−1^. These vectors were colored using MATLAB.^[^ ^32^ ^]^ Velocity fields were then averaged over time to derive the mean field on the sample within the specified time interval. Real‐time video recordings of the experiments clearly show that the flows were much faster than the calculated values. This discrepancy arises because the velocity fields were determined by detecting and tracking buoyant cavitation bubbles, which vary in size and were carried by high‐speed convective flows. These bubbles also move rapidly in and out of the focal plane, making it prohibitively difficult to track them consistently. Since the bubbles do not fully capture the fluid flow dynamics, the actual fluid velocities were expected to be significantly higher than the average velocities calculated from bubble movement (see also Supporting Information).

### Temperature Profiling of the Suspension

The suspension temperature was measured using a K‐type thermocouple probe inserted in the liquid during a typical synthesis. The temperature values were measured using a digital multimeter (Protek 506) connected to a computer to record the measurements for 20 min The laser beam was blocked within the first 2 min of the measurement.

### List of Chemicals Used in this Work

1. Aluminium isopropoxide (Al(iPro)_3_, > 98%, 220 418, Aldrich),
2. Al powder (325 mesh, 99.5%, Alfa Aesar),
3. Deionized water (DI H_2_O, resistivity = 18.2 MΩ),
4. Ethanol (EtOH, 99.99%, IsoLab),
5. Sodium hydroxide (NaOH, 99%, Merck),
6. Tetraethyl orthosilicate (TEOS, 98%, 131 903, Aldrich),
7. Tetramethylammonium hydroxide (TMAOH, 25 wt.%, Aldrich),
8. Tetrapropylammonium hydroxide (TPAOH, 1 M, 254 533, Aldrich),
9. Cetyltrimethylammonium bromide (CTAB, 98%, Fisher Chemical),
10. SiO_2_ (Ludox‐HS 30, 30 wt.% SiO_2_, pH = 9.8, Aldrich), and
11. Polystyrene spheres (Micro Particles GmbH, diameter: 250 nm & 500 nm, 2 wt.%).

### Synthesis Procedures—TPA‐Silicalite‐1 Zeolite (MFI type)

Three different molar formulas^[^ ^33^, ^34^ ^]^ were used for MFI‐type TPA‐Silicalite‐1 zeolite synthesis:

M1 = 25 SiO2 ∶ 9 TPAOH ∶ 1450 H2O ∶ 100 EtOH
M2 = 25 SiO2 ∶ 9 TPAOH ∶ 480 H2O ∶ 100 EtOH
M3 = 25 SiO2 ∶ 9 TPAOH ∶ 450 H2O ∶ 100 EtOH

The M1 formula was primarily used, while the M2 and M3 formulas to demonstrate their potential in reducing average crystal sizes by minimizing water content. To prepare the precursor suspension, TPAOH (0.25 g) was combined with TEOS (0.142 g) in a bottle and stirred at room temperature (RT) for 30 min Subsequently, DI water (0.512 g) was added to this solution and stirred at RT for 24 h. The same synthesis procedure was applied to the M2 and M3 molar formulas, with DI water (0.07 g) utilized for M2 and no additional water for M3. Following preparation, the solution was divided into two batches for ultrafast laser and hydrothermal synthesis to compare the resulting zeolite crystals. Hydrothermal syntheses were conducted according to recommended procedures. Hydrothermal synthesis for batches that use M1 molar formula was conducted at 100 °C in an oven (Binder FD 115) for 48 h. These conditions changed to 90 °C heating for 30 h according to synthesis requirements using M2 and M3 molar formulas. Powder products were collected following centrifugation (14 000 rpm, Eppendorf Centrifuge 5420), washed with DI water until reaching a neutral pH, and dried overnight at 45 °C in the oven.

### Synthesis Procedures—Zeolite A (LTA type)

Al(iPro)_3_ ∶ 3 TEOS ∶ 7.36 TMAOH ∶ 0.33 NaOH ∶ 192.41 H_2_O molar formula was used to synthesize microporous zeolite A. TMAOH (2.17 g) was combined with DI water (0.95 g) and stirred for 20 min Subsequently, Al(iPro)_3_ (0.17 g) was gradually added to the mixture and stirred for 1 h. After this, TEOS (0.53 g) was introduced to the mixture and stirred for 2 h. Meanwhile, NaOH (0.01 g) and DI water (0.23 g) were mixed in a separate beaker and slowly added dropwise to the main mixture. The resulting blend was stirred for 15 h at RT for aging. The laser synthesis lasted 4 h, while the hydrothermal reaction was conducted in an oven at 100 °C for 8 h. Powder products were collected using the same procedure as that of TPA‐Silicalite‐1 zeolites.

### Synthesis Procedures—ZSM‐5 Zeolite (MFI type)

Al(iPro)_3_ ∶ 50 TEOS ∶ 9 TPAOH ∶ 9 NaOH ∶ 5709 H_2_O molar formula^[^ ^35^ ^]^ was used to synthesize microporous ZSM‐5 zeolites. DI water (0.42 g) was combined with TEOS (0.26 g), followed by the slow addition of Al(iPro)_3_ (0.005 g), forming Solution A. Meanwhile, in a 5 mL beaker, NaOH powder (0.009 g) was dissolved in DI water (1.92 g) and stirred for 2 min until fully dissolved. This NaOH solution (Solution B) was mixed with TPAOH (0.23 g). Solution A and Solution B were stirred at RT for 1 h. Subsequently, Solution B was added dropwise to Solution A, stirring the mixture for an additional 24 h at RT for aging. The laser synthesis lasted 6 h, while the hydrothermal reaction was conducted in an oven at 100 °C for 24 h. Powder products were collected using the same procedure as that of TPA‐Silicalite‐1 zeolites.

### Synthesis Procedures—Zeolite Y (FAU type)

9 Na_2_O ∶ 0.7 Al_2_O_3_ ∶ 10 SiO_2_ ∶ 160 H_2_O molar formula^[^ ^6^ ^]^ was used to synthesize template‐free zeolite Y. Solution A was prepared by dissolving NaOH (2 g) in DI water (4 g), to which aluminum powder (0.189 g) was gradually added. In Solution B, colloidal silica (10 g) was combined with NaOH (1.6 g) and DI water (3.4 g) to form a turbid suspension. This mixture was then subjected to an oven at 100 °C for 6 min to convert the turbid suspension into a clear one. Afterward, Solution A was added drop by drop into Solution B under vigorous stirring, with Solution B cooling down during the mixing process. The resulting clear suspension was left to age 24 h at RT. The laser synthesis procedure lasted 5 h, while the hydrothermal reaction occurred in an oven set at 100 °C for 45 h. The powder products were collected using the same procedure as that of TPA‐Silicalite‐1 zeolites.

### Synthesis Procedures—Hierarchical ZSM‐5 Zeolite with a Soft Template

Al(iPro)_3_ ∶ 50 TEOS ∶ 18 TPAOH ∶ 1.37 CTAB ∶ 171.5 EtOH ∶ 4903 HO_2_ molar formula^[^ ^35^ ^]^ was used to synthesize hierarchical ZSM‐5 zeolites using the soft template CTAB. The primary precursor suspension was aged in an oil bath at 100 °C for 44 h. CTAB, ethanol, and DI water were introduced to the primary precursor suspension. In a separate bottle, a mixture of TEOS (1.56 g) and DI water (2.59 g) was stirred at RT for 2 h, with Al(iPro)_3_ (0.03 g) added gradually. Then, TPAOH (2.76 g) was incorporated into the solution, forming Solution A, which was subjected to an oil bath at 40 °C for 2 h. The temperature was subsequently raised to 100 °C to continue stirring the first precursor suspension for an additional 44 h. Meanwhile, CTAB (0.075 g) was dissolved in DI water (8.45 g) in a different bottle and stirred at RT for 15 min to form Solution B. After 44 h of stirring, Solution B was added to Solution A after cooling to RT. The mixture was stirred in an oil bath at 80 °C for another 2 h. Finally, ethanol (1.19 g) was introduced to the final precursor suspension for further stirring at 80 °C for 2 h. The laser synthesis lasted 5 h, while the hydrothermal reaction occurred in an oven set at 150 °C for 24 h. The powder products were collected with the additional calcination step at 490 °C for 5 h using Protherm equipment with a heating rate of 5 °C min^−1^.

### Synthesis Procedures—Mesoporogen‐free Hierarchical ZSM‐5 Zeolite

Al(iPro)_3_ ∶ 50 TEOS ∶ 9 TPAOH ∶ 9 NaOH ∶ 5709 H_2_O molar formula^[^ ^35^ ^]^ was used to obtain hierarchical zeolite without using a template to obtain mesopores (*i.e*., mesoporogens). Solution A was prepared by mixing DI water (0.83 g) with TEOS (0.51 g), then by gradually adding Al(iPro)_3_ (0.01 g). The resulting mixture was stirred at RT for 1 h. Meanwhile, Solution B was prepared concurrently, consisting of NaOH powder (0.018 g) dissolved in DI water (0.96 g) for 2 min until complete dissolution, followed by adding TPAOH (0.45 g). Solution B was stirred at RT for 10 min Subsequently, Solution A was placed in an oil bath at 40 °C, and Solution B was added dropwise while stirring for 3 h at the same temperature. The oil bath temperature was then increased to 100 °C, and the solution was stirred for an additional 44 h. Afterward, the suspension was removed from the oil bath, and DI water (2.88 g) was added to achieve the final precursor suspension. This final solution was stirred at RT for 15 min The laser synthesis lasted 5 h, while the hydrothermal reaction occurred in an oven set at 150 °C for 24 h. The powder products were collected with the additional calcination step at 490 °C for 5 h using Protherm equipment with a heating rate of 5 °C min^−1^.

### Synthesis Procedures—Multiporous Hierarchical Crystals with a Hard Template

M2 molar formula of MFI‐type TPA‐Silicalite‐1 zeolites was used to synthesize hierarchical crystals. The precursor preparation and aging steps followed the procedure outlined for TPA‐Silicalite‐1 zeolite synthesis, with one key variation: adding 250 and 500 nm polystyrene (PS) nanoparticles (1.3 mg) to precursor suspension (500 µL) in separate syntheses. These PS nanoparticles, obtained in powder form, served as hard templates to induce the formation of multi‐porous topologies. To achieve the PS in powder form, the PS solution underwent centrifugation (14 000 rpm, Eppendorf Centrifuge 5420) followed by overnight drying at 45 °C in an oven. Our synthesis employed a home‐built ultrafast burst mode laser,^[^ ^34^ ^]^ with each burst (groups of pulses) lasting 200 ns, intra‐burst repetition rate of 1.6 GHz (correspondingly, 320 pulses per burst) and inter‐burst repetition rate of 200 kHz, and pulse duration of ≈300 fs. The laser synthesis process lasted 3 h, with the average laser power incident on the glass measured at 3 W. The powder products were collected with the addition of a calcination step performed at 500 °C for 5 h using Protherm equipment with a heating rate of 5 °C min^−1^.

### Characterizations

The crystallographic structure of the zeolite powder samples was analyzed using an XRD spectrometer (Malvern Panalytical X'Pert Pro Multi‐purpose Diffractometer), with a Cu *K_α_* X‐ray source (*K_α_* = 1.54187 Å) operating at 45 kV and 40 mA, scanning between the 2θ angle range of 5°–50°. HR‐TEM images and SAED patterns were obtained using a FEI Tecnai G2 20 S Twin microscope operating at 200 kV. SEM micrographs were obtained using an FEI Quanta 200F microscope operating at 30 kV after coating the samples with a 10 nm Au/Pd conductive layer using a Gatan 682 Precision Etching Coating system. FEI FP2067/30, equipped with a through‐lens detector in immersion mode, was used for the FIB‐SEM micrographs with higher magnifications.

Particle size distribution analyses were conducted using ImageJ and Origin(Pro) software, based on a minimum of four SEM images of the same sample taken from various locations along the substrate. A fixed number of crystals (298) were utilized for the analyses, with the analysis‐statistics toolbox of Origin employed to generate histograms depicting the average size distribution. The results were presented as mean ± standard deviation (SD), and each form of zeolite synthesis was carried out separately at least five times. The figure captions provide the sample sizes for each particle size distribution.

The surface area and pore size distribution were evaluated through BET N_2_ adsorption‐desorption isotherms at 77.3 K utilizing the Quantachrome Autosorb IQ2 MP gas sorption system. A total of 18 synthesis cycles were conducted to obtain the requisite 150 mg powder sample. Before BET measurements, the powder sample underwent outgassing at 300 °C for 16 h. The BET method facilitated the determination of the total surface area, while the micropore volume and the pore size distribution were calculated via DFT.

Thermal stability, zeolitic water, and structure‐directing agent (SDA) content were tested using a TGA spectrometer (SDT650 TGA‐DSC) under airflow within 25 to 900 °C temperature range with a 5 °C min^−1^ of heat flow rate. The DTA curve was obtained from the TGA data via differentiation using Origin software.

The crystallinity index (%) of the products was calculated using XRD data from 2 mg powder samples. After subtracting the baseline from the XRD spectrum, the peak areas (PA) of the most significant peaks (e.g., (501), (051), (151), (303), and (133) planes within the 22° to 25° 2θ region for TPA‐Silica‐1 zeolites) were calculated and summed using Origin software. Following the methodology outlined in the references,^[^ ^36^, ^37^ ^]^ the crystallinity index (*α*) was calculated with respect to a reference that has the maximum achievable crystallinity:

(1) $$\propto \left(\%\right)=\frac{\sum PA_{laser}}{\sum PA_{reference}}\;\cdot 100$$

The crystallinity calculations from liquid samples were conducted using ATR‐FTIR spectroscopy (Bruker Vertex 70 V). Peak deconvolution analyses were performed using a custom MATLAB function.^[^ ^38^ ^]^ Prior to deconvolution, baseline correction was applied to the IR and XRD data using the asymmetric least squares algorithm implemented in Origin(Pro) software. The MFI precursor (brown‐filled peak) and fully‐grown crystal (grey‐filled peak) were considered to calculate the IR crystallinity index. The sums were then normalized, and the crystallinity index graph shown in Figure 5C was fit to a 4‐parameter logistic function

(2) $$f\;\left(x\right)=d+\frac{a-d}{1+{\left(\frac{x}{c}\right)}^{b}}$$

where *x* denotes the time, *a* and *d* are the minimum and maximum values that can be obtained, respectively. *c* is the point of inflection (i.e., the point on the S‐shaped curve halfway between *a* and *d*). *b* is the Hill's slope of the curve (i.e., this was related to the curve's steepness at point *c*).

The product yield was calculated by dividing the powder product weight by that of SiO_2_ in the batch suspension. The measurement of the powder product was conducted post‐centrifugation and drying. The calculated water and TPA contents obtained from TGA‐DTA were deducted from the total mass. The batch SiO_2_ weight was computed based on the molar formula.

## Conflict of Interest

The authors declare no conflict of interest.

## Author Contributions

S.G. and M.H. contributed equally to this work. S.G. and S.I. designed the research. S.G., M.H., and M.M.D. performed the experiments and characterizations. S.G., M.H., M.M.D., Ü.S.N., and S.I. analyzed the data. Ö.Y., G.M., M.L., and A.C. helped design the experimental setup. Ö.Y. and F.Ö.I. provided the energy calculations. B.A.K. provided facility access for some critical instruments and helped with those experiments. P.A. built the home‐built ultrafast burst‐mode laser. S.I. wrote the paper and supervised the study. All authors read and discussed the final manuscript.

## Supporting information



Supporting Information

Supplemental Video 1

Supplemental Video 2

Supplemental Video 3

Supplemental Video 4

## Data Availability

The data that support the findings of this study are available from the corresponding author upon reasonable request.

## References

[1] Y. Li , J. Yu , Nat. Rev. Mater. 2021, 6, 1156.

[2] R. L. Siegelman , E. J. Kim , J. R. Long , Nat. Mater. 2021, 20, 1060. 34321657 10.1038/s41563-021-01054-8

[3] E. T. C. Vogt , B. M. Weckhuysen , Chem. Soc. Rev. 2015, 44, 7342. 26382875 10.1039/c5cs00376h PMC4594121

[4] C. S. Cundy , P. A. Cox , Chem. Rev. 2003, 103, 663. 12630849 10.1021/cr020060i

[5] W. Chaikittisilp , T. Okubo , Science 2021, 374, 257. 34648342 10.1126/science.abm0089

[6] H. Awala , J. Gilson , R. Retoux , P. Boullay , J. Goupil , V. Valtchev , S. Mintova , Nat. Mater. 2015, 14, 447. 25559425 10.1038/nmat4173

[7] E. Ng , D. Chateigner , T. Bein , V. Valtchev , S. Mintova , Science 2012, 335, 70. 22157080 10.1126/science.1214798

[8] Z. Liu , J. Zhu , T. Wakihara , T. Okubo , Inorg. Chem. Front. 2019, 6, 14.

[9] M. Nüchter , B. Ondruschka , W. Bonrath , A. Gum , Green Chem. 2004, 6, 128.

[10] G. A. Tompsett , W. C. Conner , K. S. Yngvesson , Chem. Phys. Chem. 2006, 7, 296. 16463324 10.1002/cphc.200500449

[11] X. Chen , M. Qiu , S. Li , C. Yang , L. Shi , S. Zhou , G. Yu , L. Ge , X. Yu , Z. Liu , N. Sun , K. Zhang , H. Wang , M. Wang , L. Zhong , Y. Sun , Angew. Chem.–Int. Ed. 2020, 59, 11325. 10.1002/anie.202002886 32232925

[12] J. Chen , M. Zhang , J. Shu , M. Yuan , W. Yan , P. Bai , L. He , N. Shen , S. Gong , D. Zhang , J. Li , J. Hu , R. Li , G. Wu , Z. Chai , J. Yu , S. Wang , Angew. Chem.–Int. Ed. 2021, 60, 14858. 10.1002/anie.202103766 33851777

[13] G. Feng , P. Cheng , W. Yan , M. Borona , X. Li , J. H. Su , J. Wang , Y. Li , A. Corma , R. Xu , J. Yu , Science 2016, 351, 1188. 26965626 10.1126/science.aaf1559

[14] J. P. Longtint , C.‐L. Tien , Int. J. Heat Mass Transfer 1997, 40, 951.

[15] J. Noack , A. Vogel , IEEE J. Quant. Electron. 1999, 35, 1156.

[16] S. Ilday , G. Makey , G. B. Akguc , Ö. Yavuz , O. Tokel , I. Pavlov , O. Gülseren , F. Ö. Ilday , Nat. Commun. 2017, 8, 14942. 28443636 10.1038/ncomms14942 PMC5414064

[17] G. Makey , S. Galioglu , R. Ghaffari , E. D. Engin , G. Yıldırım , Ö. Yavuz , O. Bektaş , S. Nizam , Ö. Akbulut , Ö. Şahin , K. Güngör , D. Dede , H. V. Demir , F. Ö. Ilday , S. Ilday , Nat. Phys. 2020, 16, 795.

[18] K. Eidmann , J. Meyer‐Ter‐Vehn , T. Schlegel , S. Hü , Phys. Rev. E. 2000, 62, 1202. 10.1103/physreve.62.1202 11088579

[19] A. L. Gaeta , Phys. Rev. Lett. 2000, 84, 3582. 11019151 10.1103/PhysRevLett.84.3582

[20] L. Franzel , M. F. Bertino , Z. J. Huba , E. E. Carpenter , Appl. Surf. Sci. 2012, 261, 332.

[21] M. Navarro , Ú. Mayoral , E. Mateo , R. Lahoz , G. F. De La Fuente , J. Coronas , Chem. Phys. Chem. 2012, 13, 736. 22266775 10.1002/cphc.201100783

[22] X. Li , Z. Wang , J. Zheng , S. Shao , Y. Wang , Y. Yan , Chin. J. Catal. 2011, 32, 217.

[23] Q. Ge , J. Shao , Z. Wang , Y. Yan , Micropor. Mesopor. Mater. 2012, 151, 303.

[24] M. Mazur , P. S. Wheatley , M. Navarro , W. J. Roth , M. Položij , A. Mayoral , P. Eliášová , P. Nachtigall , J. Čejka , R. E. Morris , Nat. Chem. 2016, 8, 58. 26673264 10.1038/nchem.2374

[25] H. Dai , Y. Shen , T. Yang , C. Lee , D. Fu , A. Agarwal , T. T. Le , M. Tsapatsis , J. C. Palmer , B. M. Weckhuysen , P. J. Dauenhauer , X. Zou , J. D. Rimer , F. catalysts , Nat. Mater. 2020, 19, 1074. 32778812 10.1038/s41563-020-0753-1

[26] M. Razavian , S. Fatemi , M. Masoudi‐Nejad , Adsorpt. Sci. Technol. 2014, 32, 73.

[27] L. Tosheva , B. Mihailova , V. Valtchev , J. Sterte , Micropor. Mesopor. Mater. 2000, 39, 91.

[28] D. Lesthaeghe , P. Vansteenkiste , T. Verstraelen , A. Ghysels , C. E. A. Kirschhock , J. A. Martens , V. Van Speybroeck , M. Waroquier , J. Phys. Chem. C 2008, 112, 9186.

[29] C. Y. Hsu , A. S. T. Chiang , R. Selvin , R. W. Thompson , J. Phys. Chem. B 2005, 109, 18804. 16853420 10.1021/jp0526391

[30] C. E. A. Kirschhock , R. Ravishankar , F. Verspeurt , P. J. Grobet , P. A. Jacobs , J. A. Martens , J. Phys. Chem. B 1999, 103, 4965.

[31] P. Elahi , C. Kerse , H. Hoogland , Ö. Akçaalan , D. K. Kesim , B. Çetin , R. Holzwarth , F. Ö. Ilday , B. Öktem , M. D. Aşık , S. Yavaş , H. Kalaycıoğlu , Nature 2016, 537, 84. 27409814 10.1038/nature18619

[32] J. Q. Krimmer , “Quiver magnitude dependent color in 2D and 3D, MATLAB Central File Exchange,” 2023.

[33] J. Hedlund , S. Mintova , J. Sterte , Micropor. Mesopor. Mater. 1999, 28, 185.

[34] B. J. Schoeman , J. Sterte , J. E. Otterstedt , Stud. Surf. Sci. Catal. 1994, 83, 49.

[35] Y. Zhu , Z. Hua , Y. Song , W. Wu , X. Zhou , J. Zhou , J. Shi , J. Catal. 2013, 299, 20.

[36] J. Qi , T. Zhao , X. Xu , F. Li , G. Sun , J. Porous Mater. 2011, 18, 509.

[37] K. Jiao , X. Xu , Z. Lv , J. Song , M. He , H. Gies , Micropor. Mesopor. Mater. 2016, 225, 98.

[38] T. O'Haver , “MATLAB Central File Exchange” can be found under https://www.mathworks.com/matlabcentral/fileexchange/23452‐ipf‐arg1‐arg2‐arg3‐arg4, (accessed: April 2024).

