# Supplementary material for: Ultrafast Laser Synthesis of Zeolites
Source: Adv Mater. 2025 Apr 17;37(29):2415562. doi: 10.1002/adma.202415562 (PMC12288796; doi:10.1002/adma.202415562)
Supplement: Supplementary file 1 — Supporting Information [file ADMA-37-2415562-s001.docx]

Supporting Information

Ultrafast Laser Synthesis of Zeolites

Sezin Galioglu*, Mehdi Hagverdiyev, Meryem M. Doğan, Özgün Yavuz, Ü. Seleme Nizam, Ghaith Makey, Aladin Şura, Mesut Laçin, Burcu Akata Kurç, Parviz Elahi, F. Ömer Ilday, Serim Ilday*

**Video Captions**

**Video S1.** Real-time video recording of an experiment demonstrating the color change of the precursor solution from transparent to opaque white.

**Video S2**. Real-time video recordings (2^nd^ row) and vector field analyses of flow patterns (3^rd^ row) for three experiments (1^st^ row), where the laser beam is focused at three different locations: the glass-liquid interface (left), a position near the interface (middle), and closer to the center of the bulk liquid (right).

**Video S3.** Real-time video recording of an experiment where the laser beam is positioned away from the glass-liquid interface so that it does not promote multiphoton absorption. As a result, there are no laser-induced high-speed convective flows, bubble formation, or plasma generation. Instead, the solution is agitated using a magnetic stirrer.

**Video S4.** Real-time video recording of the experiment where the laser synthesis is halted and resumed multiple times.

**Estimation of the energy absorption**

We begin by measuring the transmitted power and calculating the optical reflection and absorption losses of the laser beam at the various optical interfaces, from which we determine the energy deposited in the liquid (**Figure S1**).


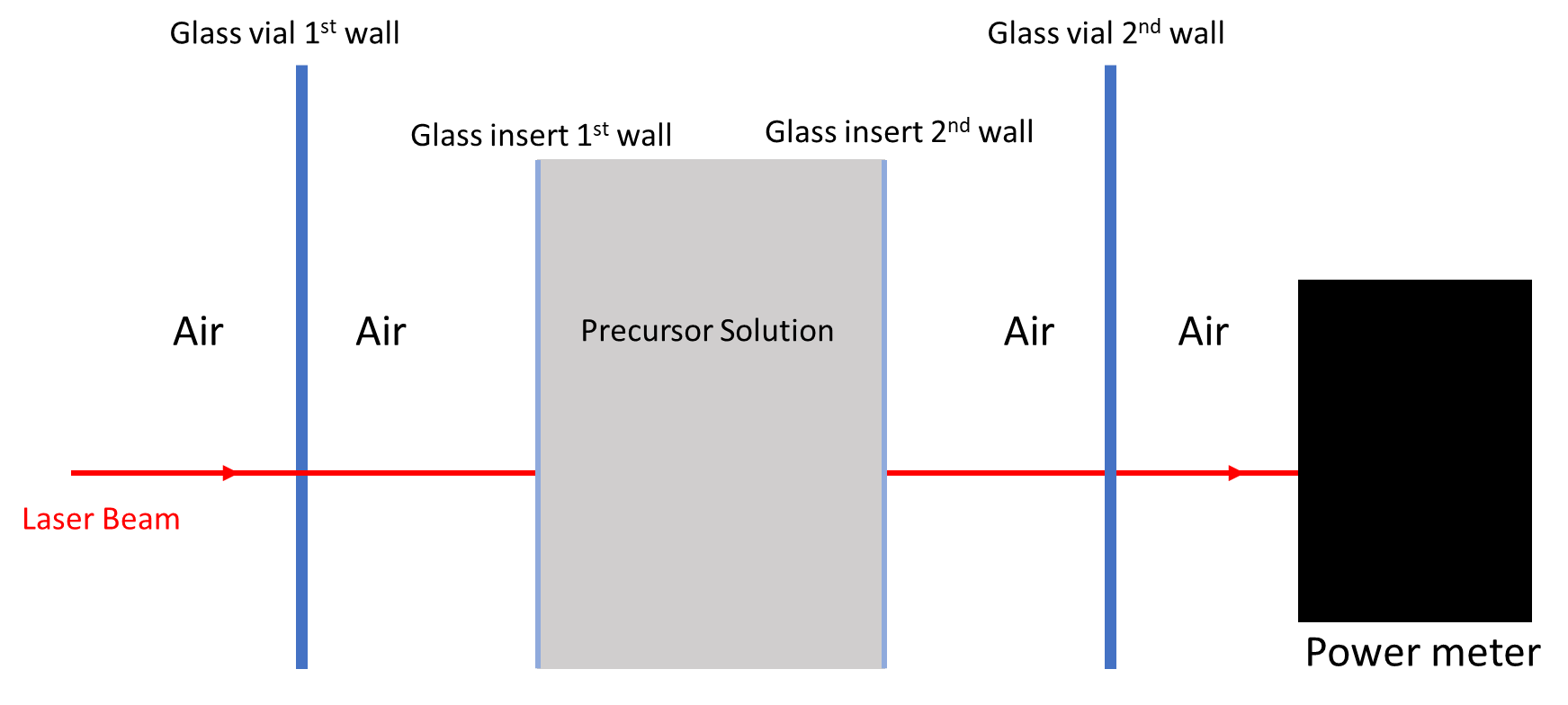


**Figure S1.** Schematic illustrating the primary optical loss mechanisms resulting from multiple interfaces and media.

**Figure S2** shows the interfaces where we measured light transmittance from the air–glass vial–air (T_1_ and T_2_), air–glass insert–air (T_3_ and T_4_), and glass insert–precursor solution–glass insert (T_5_ and T_6_) interfaces. We calculated T_1_ and T_2_ by measuring the light passing through an empty glass vial. Similarly, T_3_ and T_4_ were measured using an empty glass insert. The glass insert and vial wall thicknesses were measured as 0.92 mm and 1.16 mm.


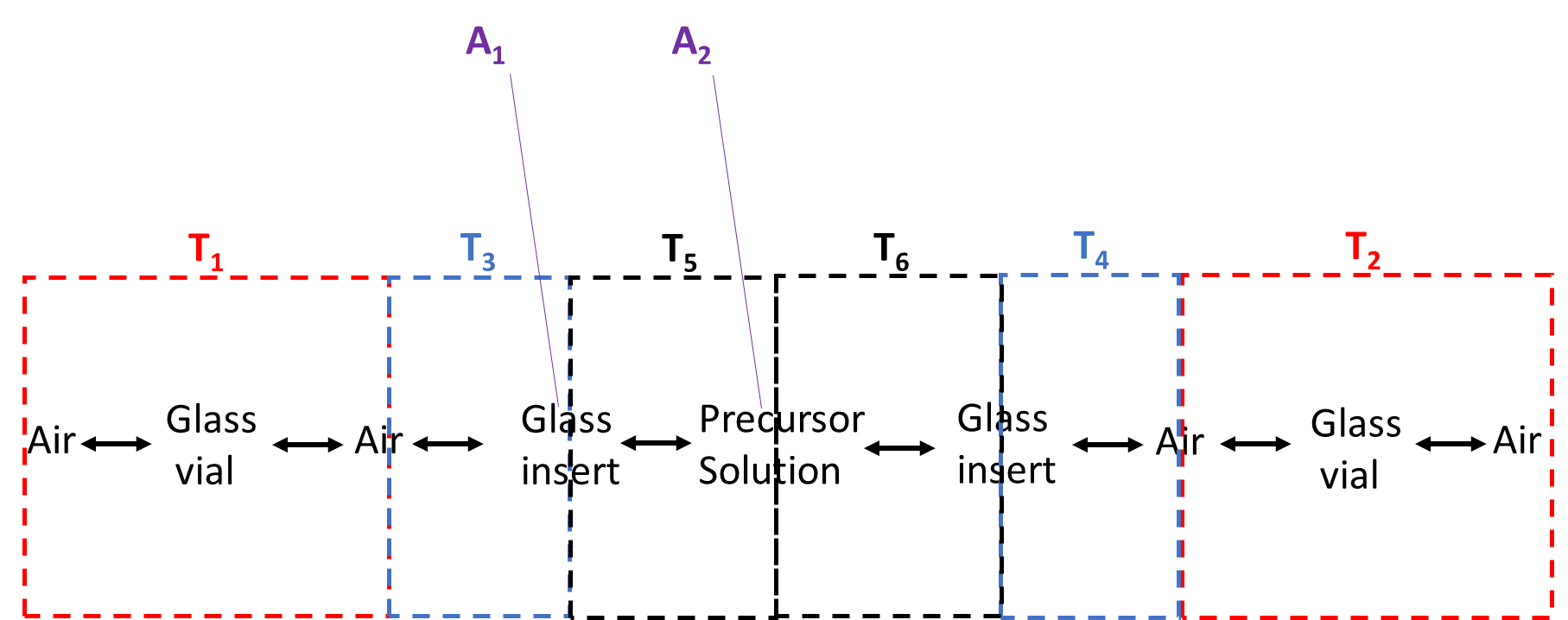


**Figure S2.** Schematic illustrating the types of measurements taken at each interface.

Next, the losses due to reflection and absorption by the 1^st^ wall of the glass insert (A1) and the precursor solution (A2) were measured by filling the glass insert with the precursor solution. During absorbance measurements, the transmitted power naturally fluctuated due to the formation of plasma, fluid flows, and bubbles. Therefore, we measured the average value. Furthermore, we assumed that the refractive index of the precursor solution was similar to that of the water. Based on the experimental measurements, we determined that 22.5% of the incident average laser power is absorbed within the precursor solution.

**Calculation of the thermal gradients**

We will next estimate the magnitude of the thermal gradients formed around the beam focus. Due to the presence of multiple nonlinear dissipative and scattering mechanisms, such as multi-photon absorption, plasma and cavitation bubble formation, and convective flows, an accurate calculation is prohibitively difficult. However, using the measured absorbed power and pulse parameters and aided by a set of reasonable approximations, we can calculate an approximate value, likely a lower bound.

Given the empirically determined absorption of 22.5% of the laser power in the solution, we first calculate the time it should take to heat the entire liquid from room temperature of 21 °C to 95 °C, the steady state average temperature, ${\Delta T}_{\mathrm{ave}}$, measured directly in the experiments. For this calculation, we assume the liquid to be pure water, and we neglect other dissipative processes, such as nucleation and crystal growth, bubble generation, plasma formation, and convective flows. For an incident laser power of 6 W, the absorbed power is, $P_{\mathrm{abs}}=1.35$ W. Then, the time to raise the temperature of the bulk liquid to its steady state value is given by

$\Delta t_{\mathrm{bulk}}=\frac{m_{\mathrm{tot}}C\Delta T_{\mathrm{ave}}}{P_{\mathrm{absorbed}}}\cong90 s$,

where ${\Delta T}_{\mathrm{ave}}$ is 74 °C, $C=4.186$ J g^-1^ °C^-1^ is the specific heat of water, and $m_{\mathrm{tot}}=0.08$ g is the mass of the liquid, assumed to be water, for its volume of $V_{\mathrm{total}}=80$ mm^3^. Given that we did not consider any heat losses out of the enclosure, the calculated buildup time of approximately 90 s for the bulk liquid to reach the experimentally measured temperature broadly agrees with the measured time shown in Figure 1D.

Now that we have determined the power absorbed and corresponding temperature rise on average for the entire liquid, next, we focus on the more challenging problem of estimating the same values for the laser-liquid interaction volume.

The beam is focussed to an ellipsoid with a volume ($V_{\mathrm{focal}}$), which can be calculated using the Rayleigh length ($z_{R}$) and beam waist ($\omega_{0}$) as below. The beam spot size was measured to be 9 µm using a beam profiler (Thorlabs BP209-IR2/M).

$V_{\mathrm{focal}}=\frac{4}{3}\pi{\omega_{0}}^{2}z_{R}$,

$z_{R}=\frac{\pi{\omega_{0}}^{2}}{\lambda}$,

$z_{R}=\frac{(3.14){(4.5 x {10}^{-6})}^{2}}{(1040 x {10}^{-9})}=60$ µm,

$V_{\mathrm{focal}}=\frac{4}{3}\left( 3.14 \right)\left( 4.5 \mu m \right)^{2}\left( 60 \mu m \right)=5.1\cdot{10}^{-6}$ mm^3^,

$V_{\mathrm{focal}}=5100$ µm^3^.

The liquid is almost completely transparent at the beginning of the synthesis, which means there is negligible linear absorption. Even later, when it turns opaque white, this indicates increased scattering, not absorption. Consistent with the liquid being largely water, we assume linear and even two-photon absorption at the laser wavelength of 1 µm to be negligible and that the dominant absorption mechanism, at least prior to the formation of a plasma, is three-photon absorption. Because three-photon absorption scales with the square of the peak intensity, the absorbed power for a Gaussian pulse shape and beam shape that is also Gaussian will be approximately proportional to the cube of a Gaussian ellipsoid. Integrating over such a shape, compared to linear absorption of the same Gaussian ellipsoid, the effective volume is decreased by a factor of $3^{3/2}\cong5.2$. Thus, the interaction volume, $V_{\mathrm{int}}$, over which appreciable absorption occurs, is approximately one-fifth of the focal volume, or $V_{\mathrm{int}}=980$ µm^3^. Even after establishing a plasma, which introduces linear absorption, the interaction volume is not expected to change substantially because the plasma will remain largely confined to it.

Now, we can calculate the temperature rise caused by the absorption of a single pulse. We assume three-photon absorption is dominant, thus there is negligible absorption outside of the interaction volume. We momentarily neglect all dissipative effects occurring during the pulse (within 300 fs), as well as higher-order multi-photon processes. Also, as before, we ignore all the chemicals and proceed as if the liquid is pure water. We further ignore any phase changes, such as boiling, which need a far longer timescale, during the ultrashort pulse. For the absorbed power of $P_{\mathrm{abs}}=1.35$ W and pulse repetition rate of $f_{R}=200$ kHz, the absorbed pulse energy is $E_{\mathrm{abs}}=P_{\mathrm{abs}}f_{R}^{-1}=6.75$ µJ, which becomes the heat source, $Q$. The temperature rise due to a single pulse is

$\Delta T_{\mathrm{pulse}}=\frac{Q}{m_{\mathrm{int}}C} \cong1600$°C (or K),

where ${\Delta T}_{\mathrm{pulse}}$ is the heat increment per pulse, $C=4.186$ J g^-1^ °C^-1^ is the specific heat of the water, and $m_{\mathrm{int}}=980$ pg is the water mass within the interaction volume. We further note that nearly all the energy is first coupled to the electronics, which equilibrate by transferring the energy to their atoms within several 100 fs. This peak temperature is reached only within approximately half of the beam diameter or about 2 µm due to three-photon absorption, whereas there is negligible absorption of laser light laterally further away and the temperature remains at its average value. Therefore, the temperature gradient reaches ${\sim10}^{6}$ K mm^-1^ albeit momentarily, because the interaction volume begins to cool rapidly immediately at the end of the pulse until the arrival of the next pulse within 5 µs (for a repetition rate of 200 kHz) due to various dissipative effects (Figure 1C). These effects include mechanical energy associated with the convective flows (along the axial direction of the beam), plasma and cavitation bubble formation, pressure waves, blackbody radiation, and the chemical reactions. With subsequent pulses, the peak temperatures builds up further, even though cooling will also be faster. Based on other ultrafast light-matter interactions, it is likely that peak temperatures achieved momentarily after subsequent pulses can rise as much as 8,000 K. This value is broadly consistent with the blackbody temperature corresponding to the experimentally observed blue-purple light emission. Consequently, we conclude the peak spatial thermal gradients calculated above may constitute a lower bound.

Finally, we will estimate the transit time of any chemical entering and exiting the interaction volume, *i.e.*, the tiny ultrafast reactor. This task is complicated by the difficulty to calculate the flow speeds achieved within the focal volume. The bubbles, which vary in size, are hard to monitor accurately due to imaging and tracking limitations, and their movement driven by buoyancy forces further complicates the situation. The interactions between the flows and bubbles cause light scattering, while plasma formation significantly alters the absorption dynamics. Experimentally, we only have a lower bound for the flow speeds far from the interaction region to be 1 mm s^-1^. However, the flow cross-section has expanded to 10s of micron, even 100 µm, where the lower boundary was established by tracking bubbles. At the center of the interaction volume, where the flow must be the fastest, the corresponding diameter is about 5 µm, which suggests that the flow can be in the order of 1 m s^-1^ or even higher by the continuity equation for an incompressible fluid. While this is a highly uncertain estimate, such high speeds are consistent with the observed experiment durations for the zeolite synthesises to be largely completed because this requires nearly all of the liquid volume to pass through the interaction volume at least once. Assuming top speeds of 1 m s^-1^, the time it takes to cross the length of the three-photon interaction volume, ~50 µm, is 50 µs, during which 10 pulses would be incident. Given the uncertainty in the assessment of the fluid speeds, this estimate should be taken as an order-of-magnitude assessment.

**Examples of laser-synthesized zeolites**


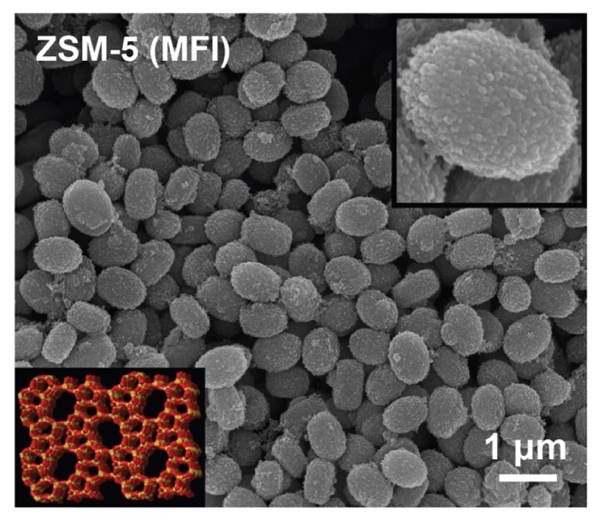

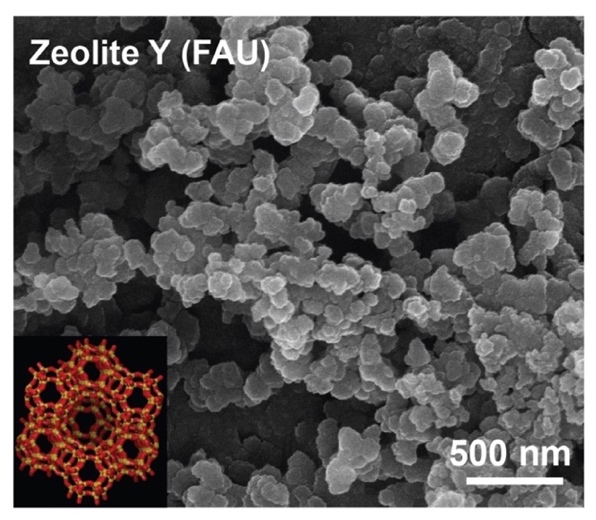


**Figure S3.** SEM images of (left) microporous ZSM-5 (MFI type) and (right) template-free Zeolite Y (FAU type) synthesized via laser synthesis method. Insets showing unit cell structures.

**
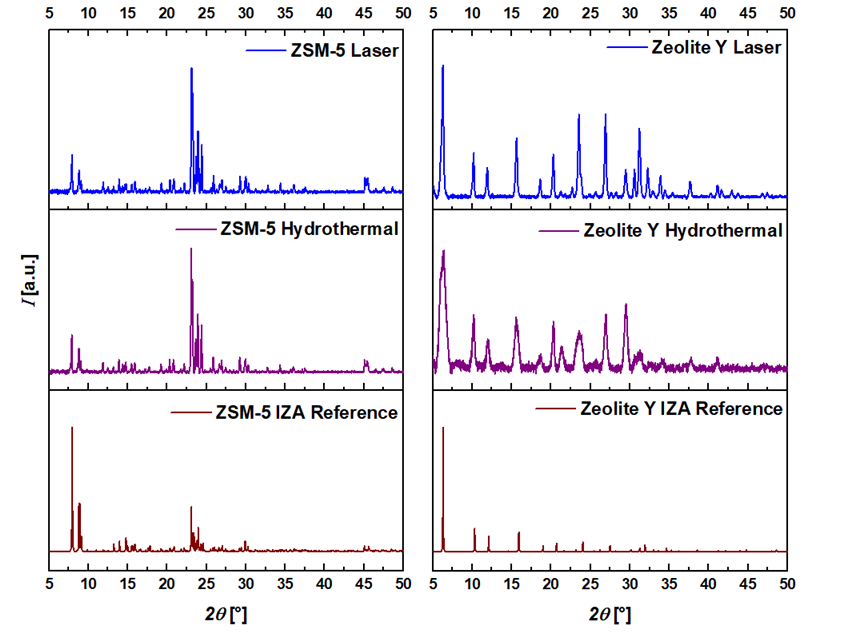
**

**Figure S4.** XRD patterns of microporous (left) ZSM-5 and (right) Zeolite Y crystals synthesized via laser and hydrothermal synthesis methods. Reference XRD patterns are obtained from the International Zeolite Association (IZA)’s webpage.

**Repeatability of the laser-synthesized zeolites**


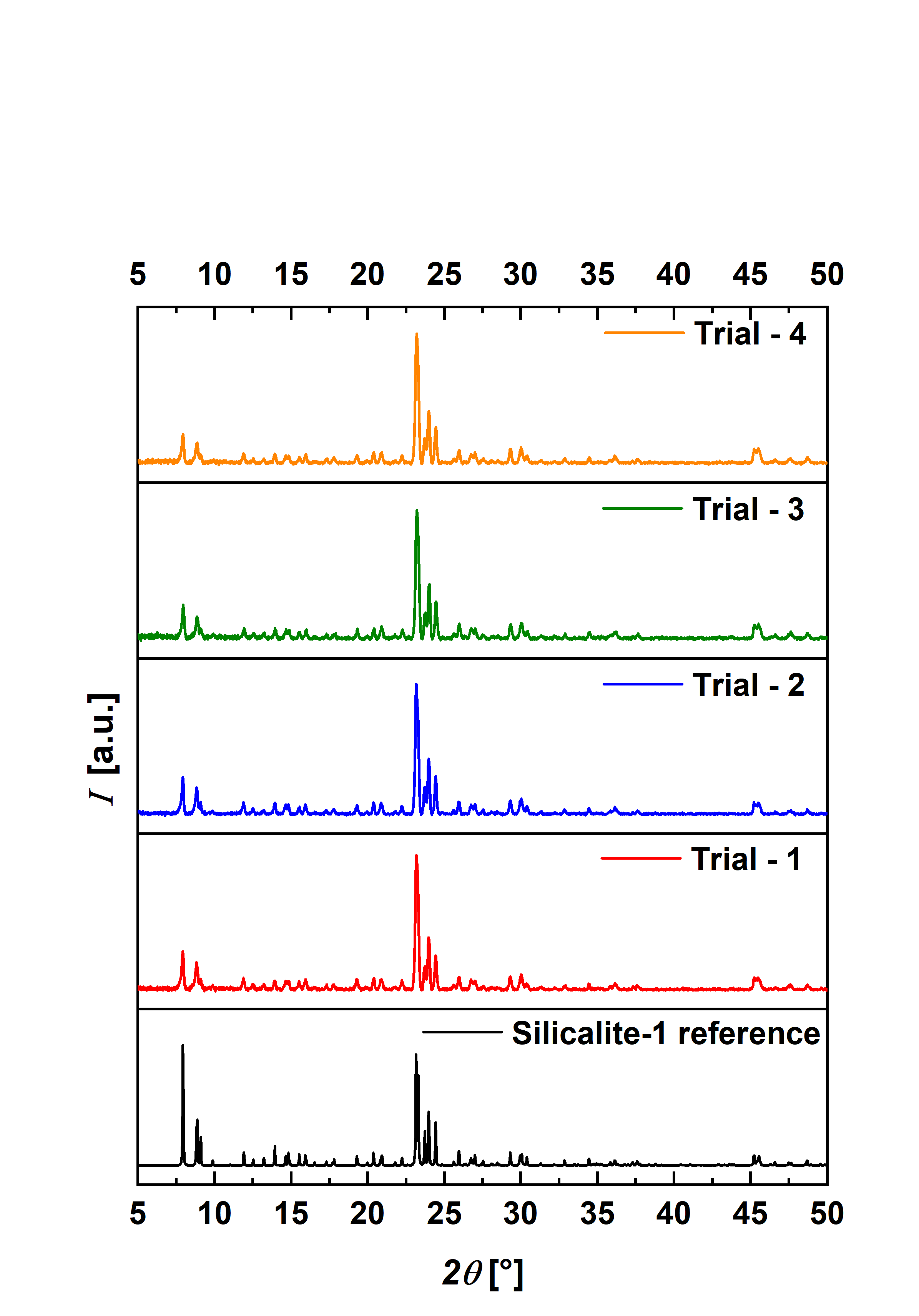


**Figure S5.** XRD patterns of laser-synthesized TPA-silicalite-1zeolites, where the original experiment (Trial 1 with 72 % crystallinity) was repeated after 2 days (Trial 2 with 86 % crystallinity), 1 week (Trial 3 with 75 % crystallinity), and 1 month (Trial 4 with 80 % crystallinity). They are compared to the reference XRD pattern approved by the International Zeolite Association (IZA).


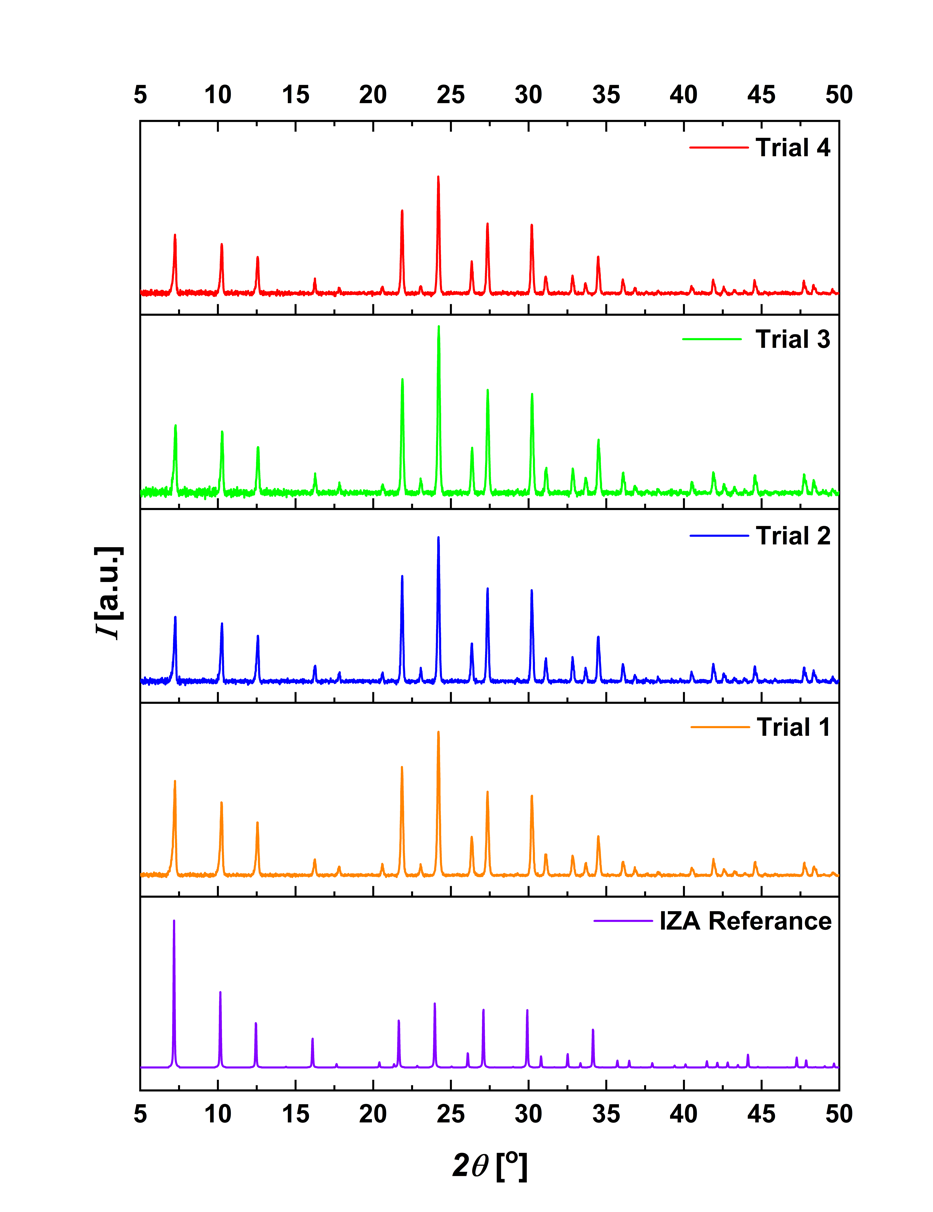


**Figure S6.** XRD patterns of laser-synthesized Zeolite A, where the original experiment (Trial 1 with 100 % crystallinity) was repeated after 1 week (Trial 2 with 97.5 % crystallinity), 1 month (Trial 3 with 93 % crystallinity) and 3 months (Trial 4 with 99 % crystallinity). They are compared to the reference XRD pattern approved by the International Zeolite Association (IZA).

**
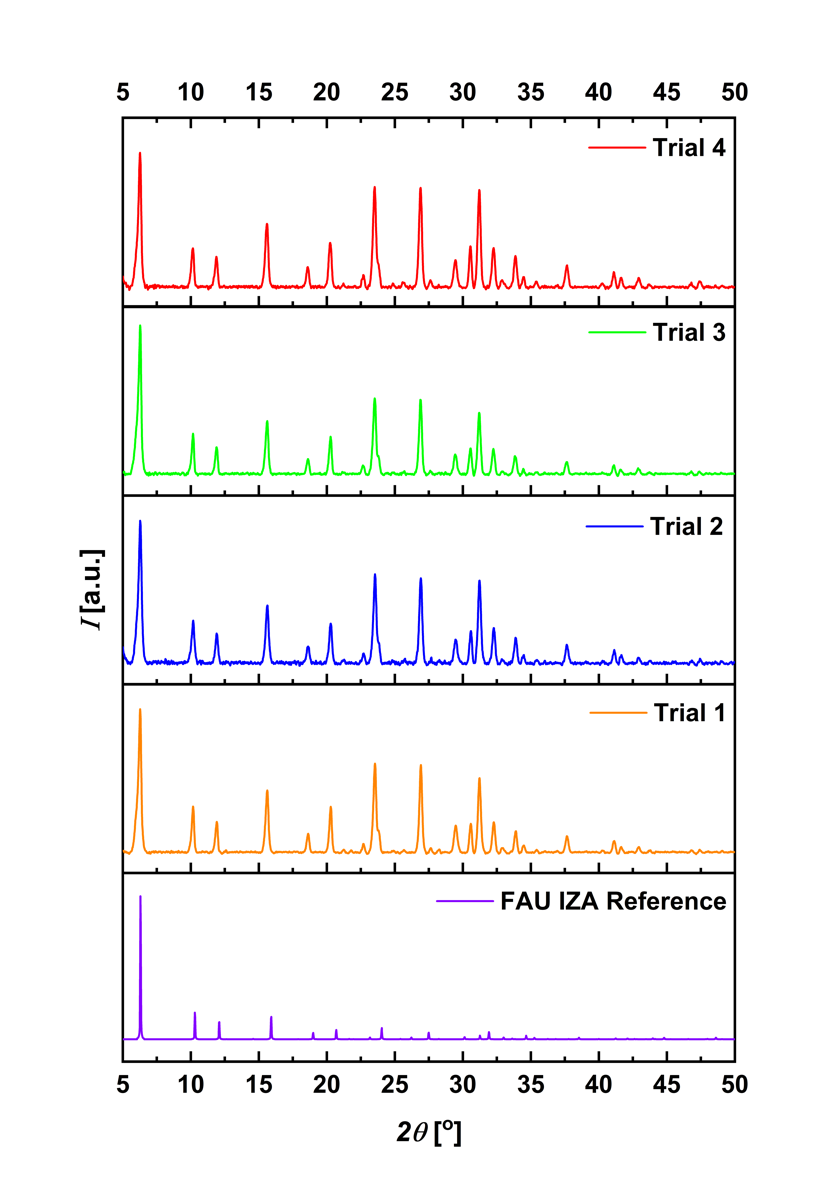
**

**Figure S7.** XRD patterns of laser-synthesized Zeolite Y, where the original experiment (Trial 1 with 96.4 % crystallinity) was repeated after 4 months (Trial 2 with 92.1 % crystallinity), 7 months (Trial 3 with 87.2 % crystallinity), and 8 months (Trial 4 with 100 % crystallinity). They are compared to the reference XRD pattern approved by the International Zeolite Association (IZA).

**Comparison of laser- and hydrothermal-synthesized TPA-Silicalite-1 zeolites**


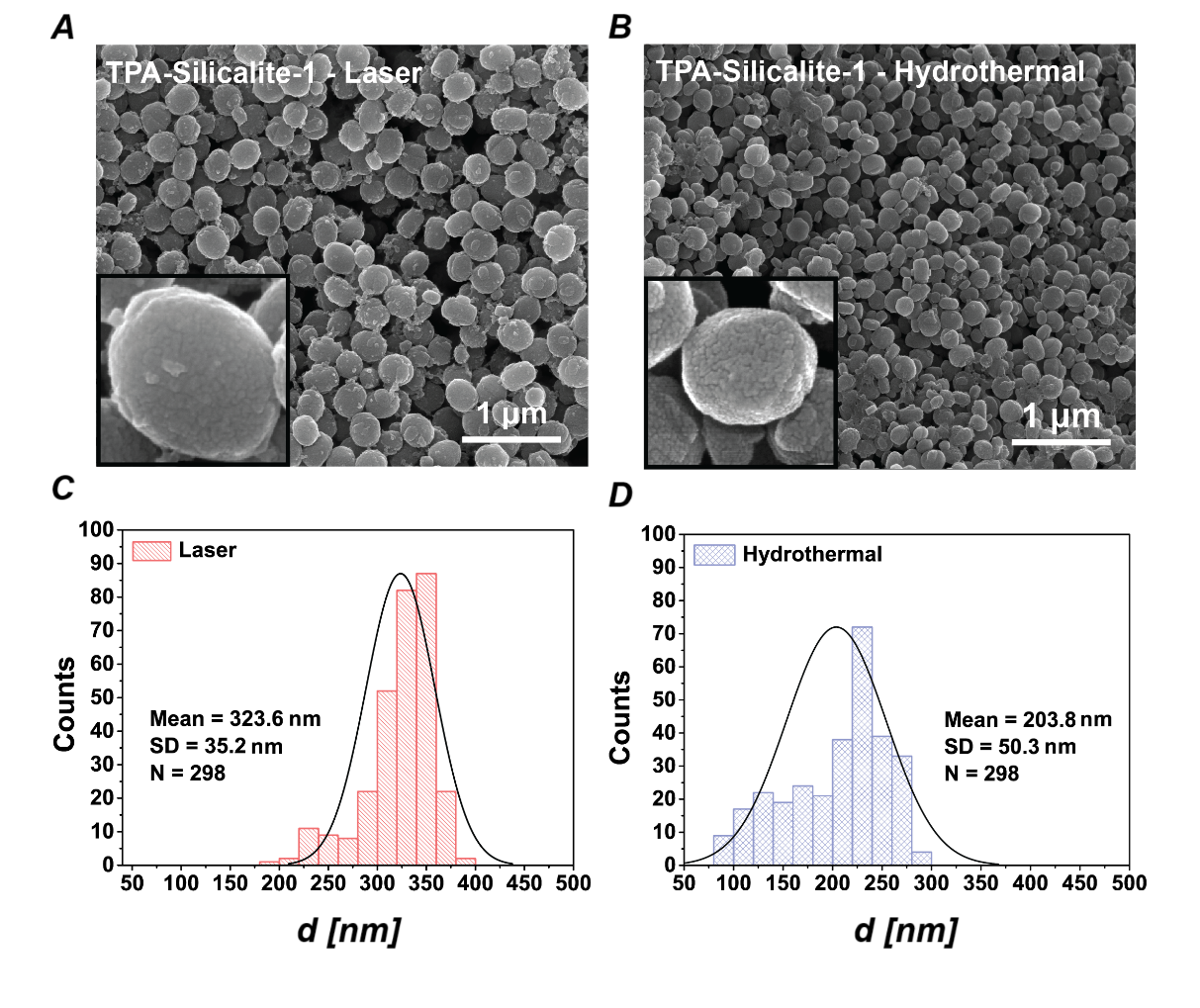


**Figure S8.** SEM images and the plots show the comparison of the particle size distribution for **(A,C)** laser- and **(B, D)** hydrothermal-synthesized zeolites.

**Reducing the average crystal size of laser-synthesized TPA-Silicalite-1 zeolites**

In previous hydrothermal synthesis trials where the effect of water content within the precursor suspension was studied, the linear growth rate of zeolite crystals seemed to increase with more water.^[1]^ Increasing the water content, all else constant, means a decrease in silica concentration and alkalinity in the synthesis mixture. Alkalinity affects number of particles and their linear growth rate (*i.e*. size). Higher alkalinity induces more number of nuclei to grow. As alkalinity increases (*i.e.,* water content decreases), particles tend to grow slower, resulting in narrower size distribution for hydrothermal and laser synthesis methods.


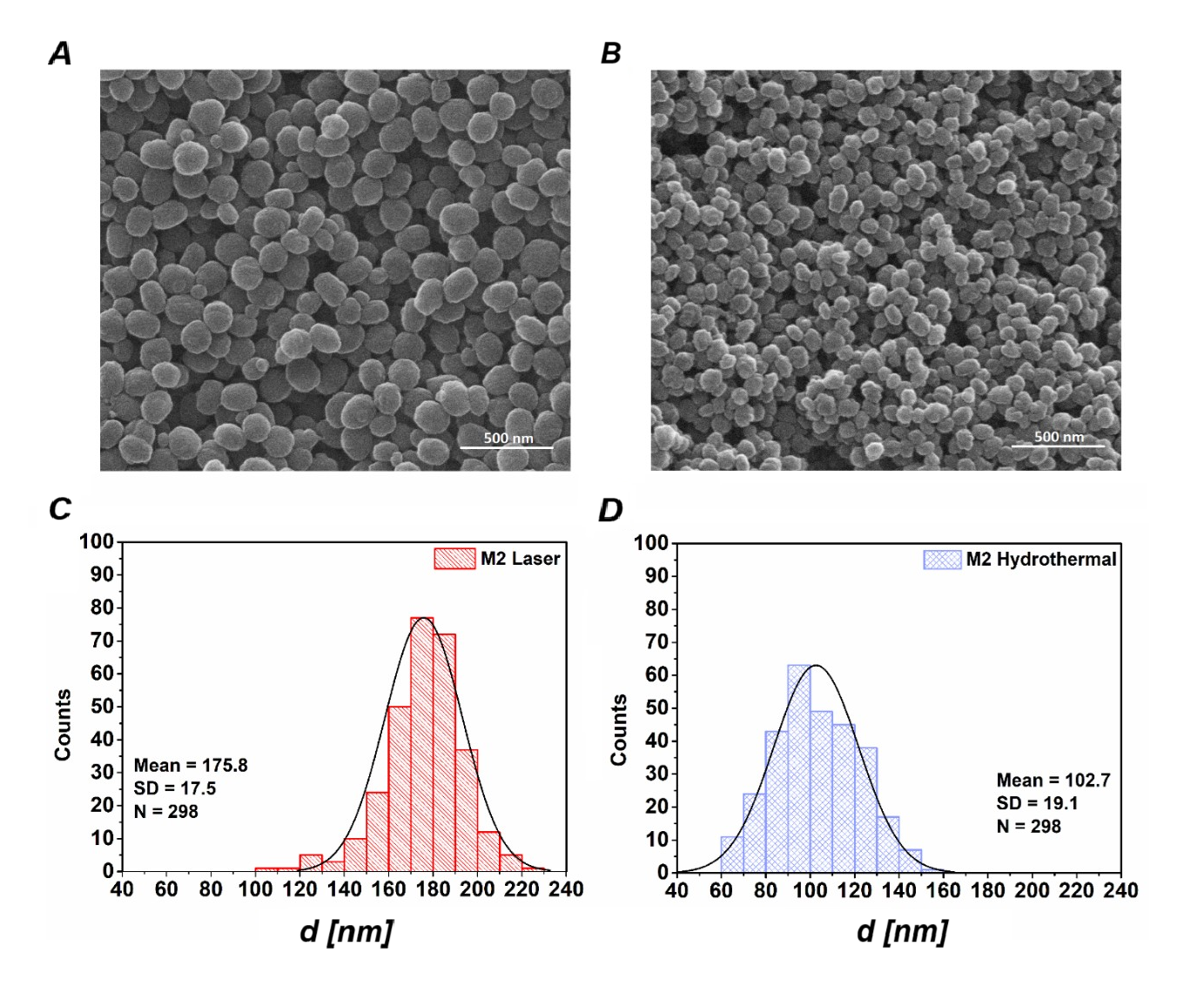


**Figure S9.** Comparison of **(A)** laser- and **(B)** hydrothermal-synthesized TPA – Silicalite-1 crystals using M2 (M2: 25 SiO_2_: 9 TPAOH: 480 H_2_O: 100 EtOH) molar formula. The rows above and below show SEM images and particle size distributions.


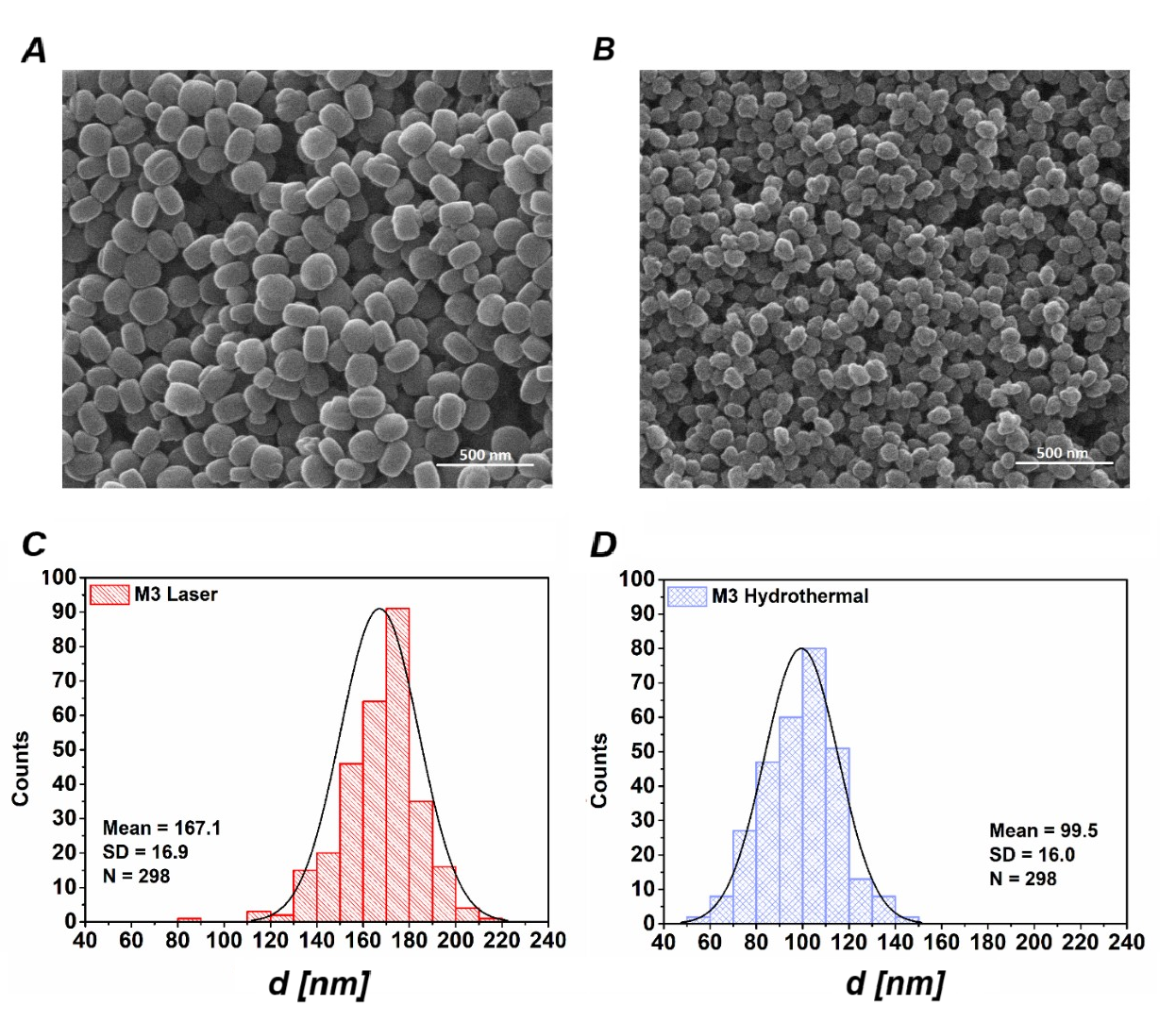


**Figure S10.** Comparison of **(a)** laser- and **(b)** hydrothermal-synthesized TPA – Silicalite-1 crystals using M3 (M3: 25 SiO_2_: 9 TPAOH: 450 H_2_O: 100 EtOH) molar formula. The rows above and below show SEM images and particle size distributions.


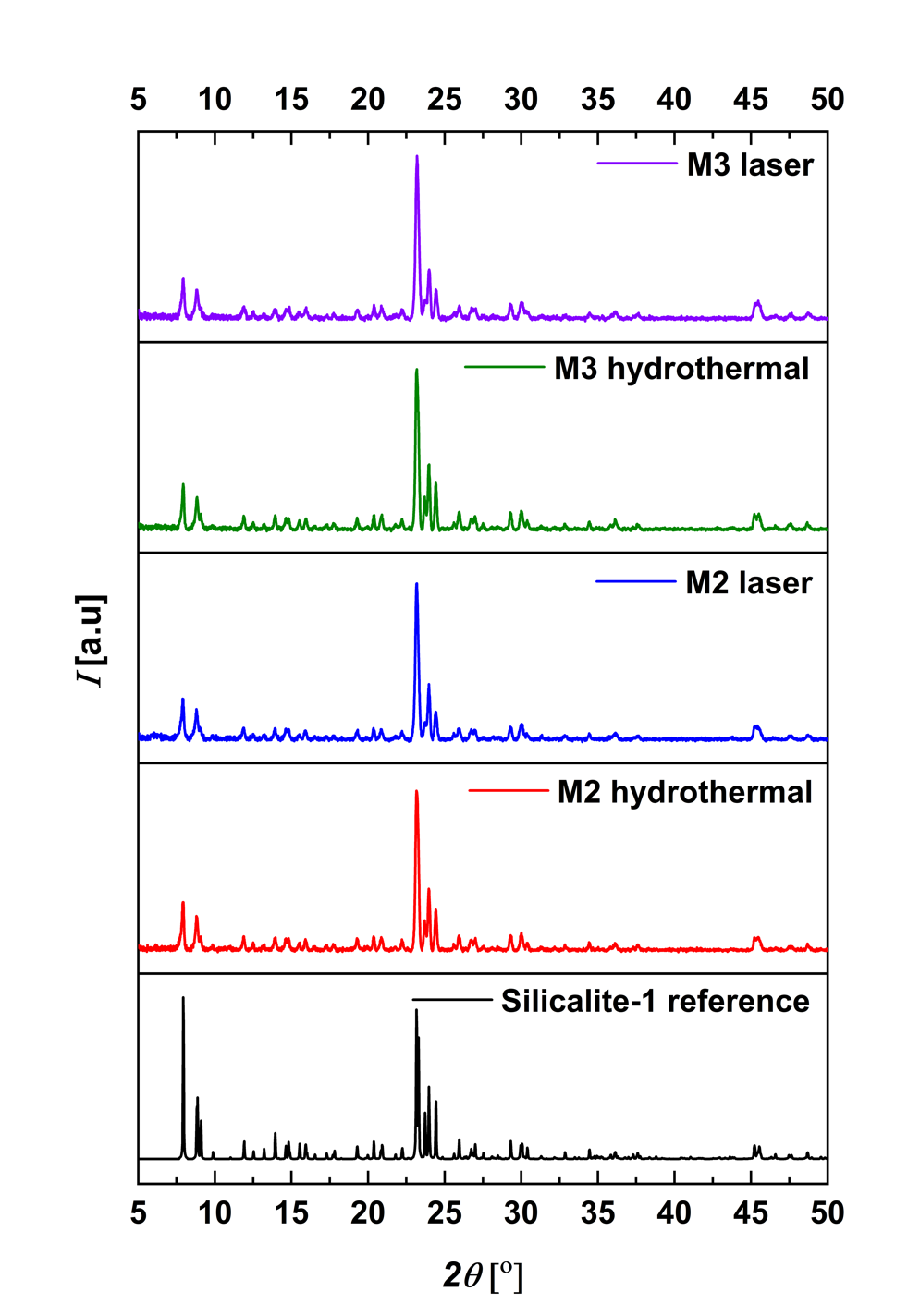


**Figure S11.** XRD spectrum of laser- and hydrothermal-synthesized TPA-Silicalite-1 zeolites using M2 and M3 molar formulas and compared to the IZA reference.

**Thermogravimetric (TGA) and Differential Thermal (DTA) analysis**

**
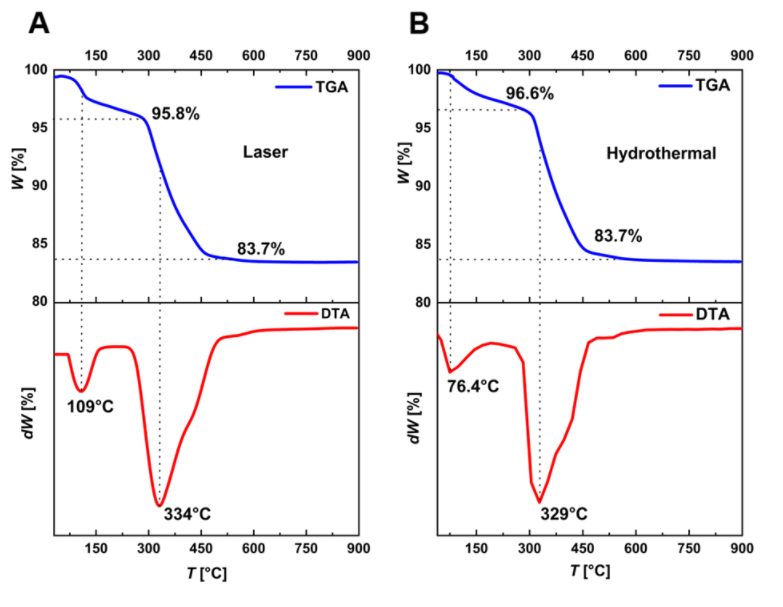
**

**Figure S12.** TGA and DTA curves of TPA-Silicalite-1 synthesized via **(A)** laser and **(B)** hydrothermal methods

**Brunauer-Emmett-Teller (BET) and Density Functional Theory (DFT) analyses**

**
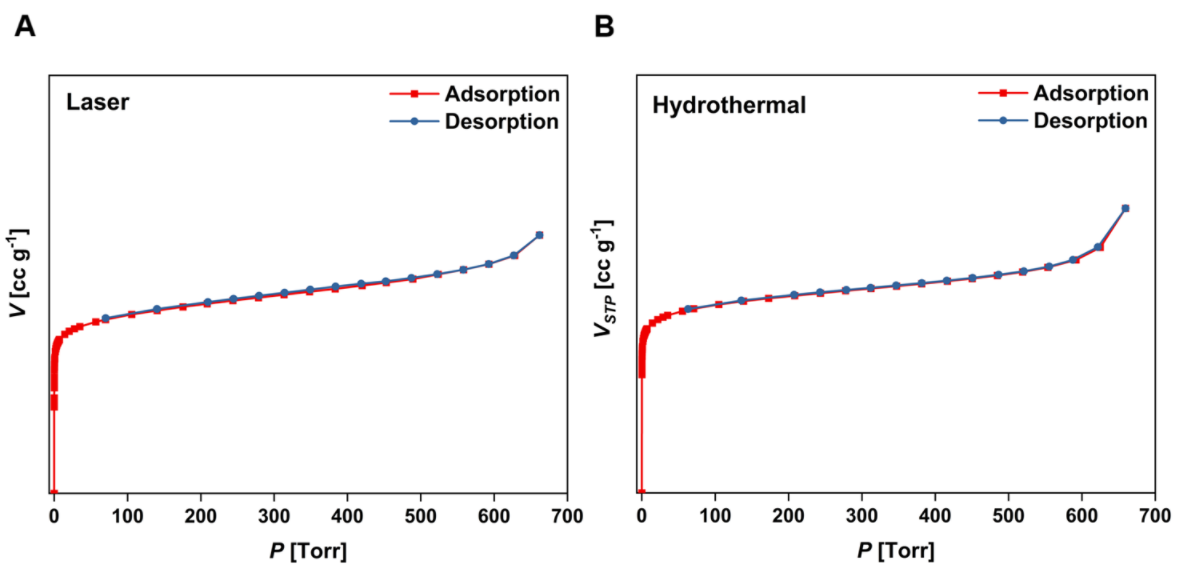
**

**Figure S13.** BET adsorption-desorption isotherms of TPA-Silicalite-1 zeolites (not calcined) synthesized with **(A)** laser and **(B)** hydrothermal methods. BET surface areas are calculated to be 335.5 m² g^-1^ for laser synthesized and 275.0 m² g^-1^ for hydrothermal synthesized crystals.

**
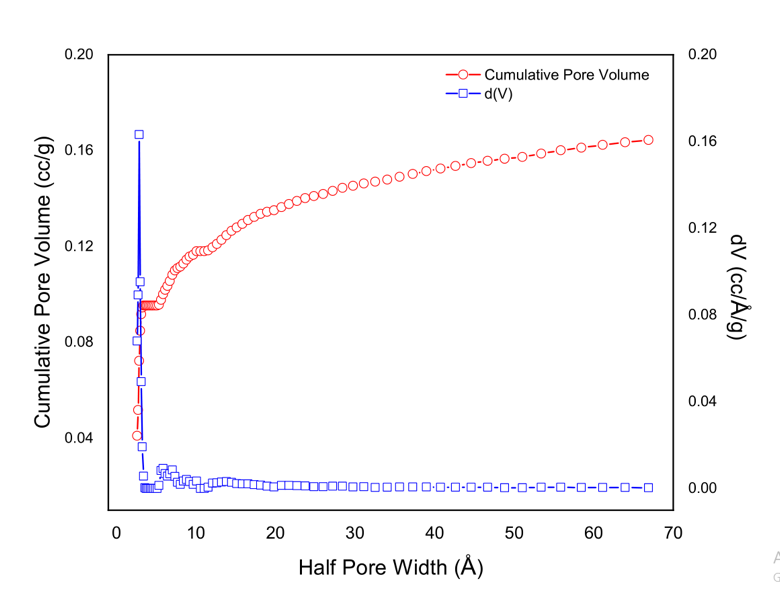
**

**Figure S14.** The cumulative pore volume and half-pore width distribution graphs for laser-synthesized TPA-Silicalite-1 zeolites. *dV* is the derivative of the cumulative pore volume with respect to pore width. The half-pore width, as determined by the DFT analysis, is 2.88 Å, and the micropore volume is 0.164 cm³ g^-1^.

**ATR-FTIR peak deconvolution analyses**


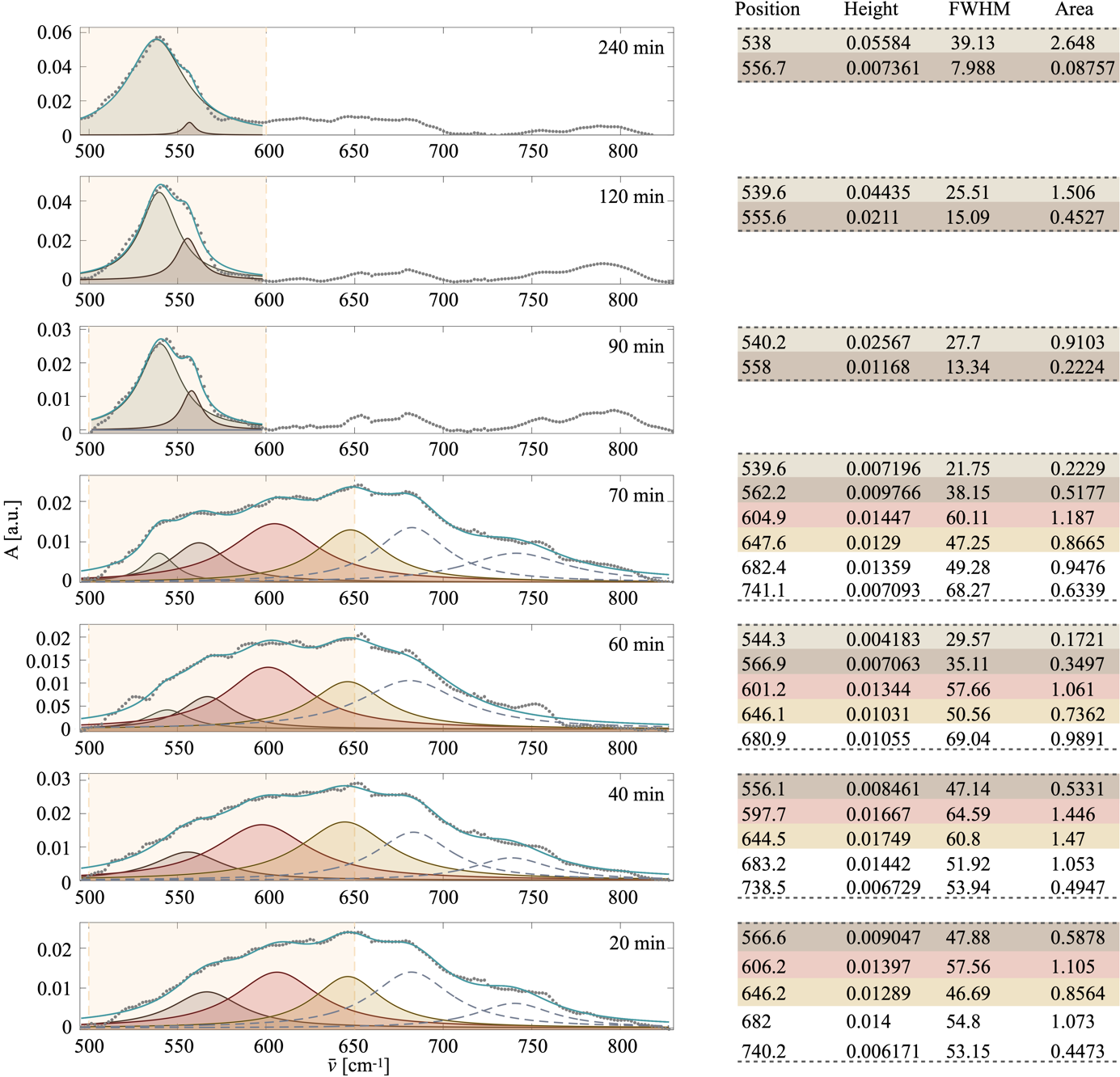


**Figure S15.** Peak deconvolution of the ATR-FTIR spectrum of laser-synthesized TPA-Silicalite-1 zeolites sampled at 20, 40, 60, 70, 90, 120, and 240 minutes of synthesis with position and height of each peak with their full-width at half maximum (FWHM) values and peak areas are color coded in the right panel.


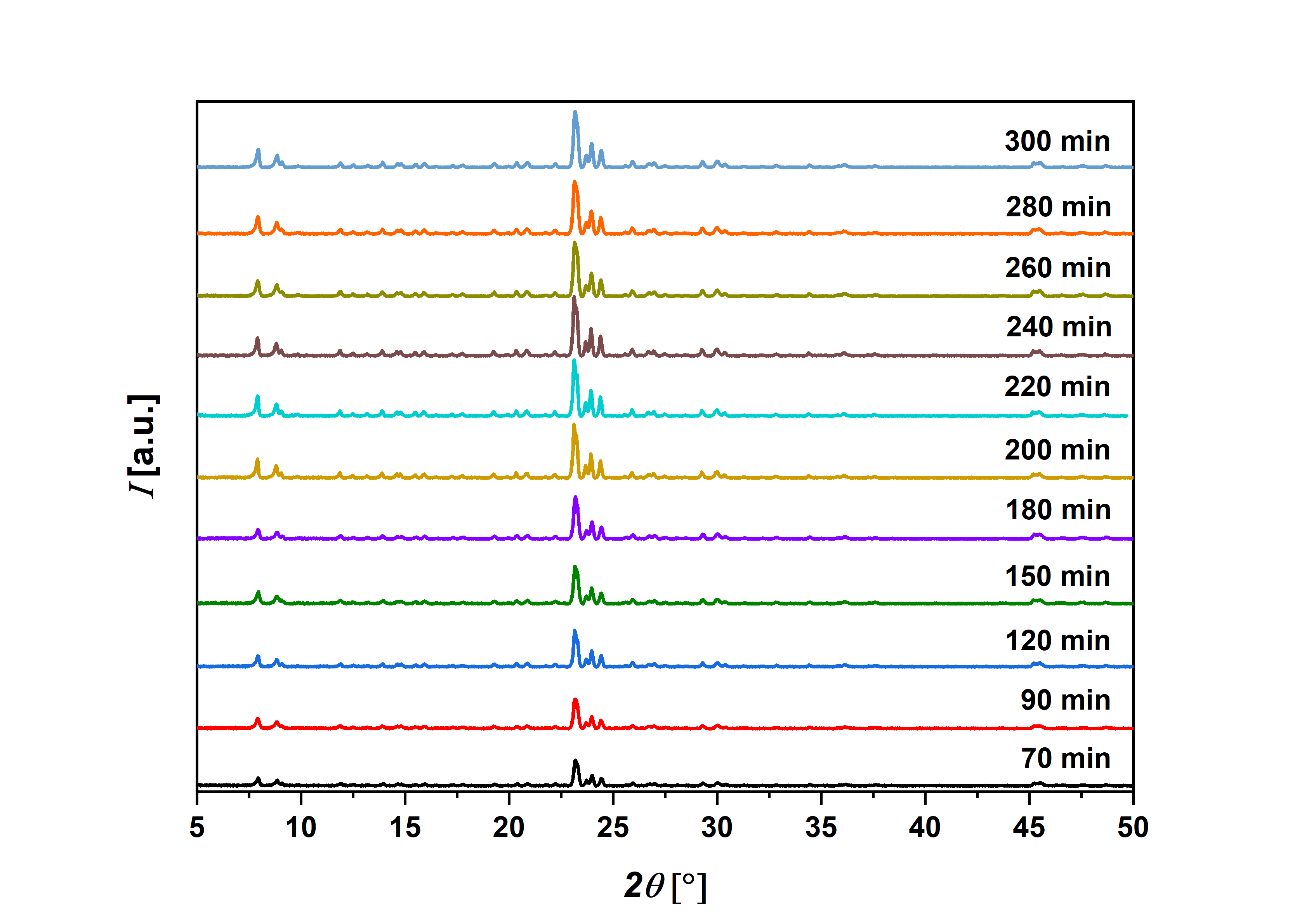


**Figure S16.** X-ray powder diffraction pattern for TPA – Silicalite-1 zeolites synthesized via ultrafast laser method applying different reaction times (see Table S3).

**Table S1.** Supplementing Figure 2, the table presents the effect of laser parameters on the crystallinity and yield of laser-synthesized TPA-Silicalite-1 zeolite crystals.

| **No** | ***V***  **[µl]** | ***P_I_* [W]** | ***P_A_*  [W]** | ***F_p_*  [J cm^-2^]** | ***E_p_*  [µJ]** | ***t***  **[h]** | ***f* [kHz]** | ***E_Tot_***  **[MJ per** $\boldsymbol{mol}_{\boldsymbol{SiO}_{\boldsymbol{2}}}$**]** | ***C***  **[%]^a),b)^** | ***Y***  **[wt.%] ^a)^** |
| --- | --- | --- | --- | --- | --- | --- | --- | --- | --- | --- |
| **T1** | 80 | 1 | 0.23 | 1.77 | 1.1 | 15 | 200 | 202.5 | - | - |
| **T2** | 80 | 1.7 | 0.38 | 1.77 | 1.1 | 9 | 333 | 202.5 | - | - |
| **T3** | 80 | 2.5 | 0.66 | 1.77 | 1.1 | 6 | 500 | 202.5 | 51 | 55.2 |
| **T4** | 80 | 5 | 1.13 | 1.77 | 1.1 | 3 | 1000 | 202.5 | 45.3 | 53.6 |
| **T5** | 80 | 2.5 | 0.56 | 0.88 | 0.6 | 6 | 1000 | 202.5 | - | - |
| **T6** | 80 | 5 | 1.13 | 3.54 | 2.3 | 3 | 500 | 202.5 | 87 | 69.7 |
| **T7** | 80 | 5 | 1.13 | 8.84 | 5.6 | 3 | 200 | 202.5 | 86 | 69.7 |

*V* - Volume, *P_I_* - incident average laser power, *P_A_* - absorbed average laser power, *F_p_* - pulse fluence, *E_p_* - pulse energy, *t* - time, *f* - frequency, *E_Tot_* - total deposited energy, *C* - crystallinity, *Y* - yield, *T* - trial.

1. The initial transparent color of the suspension did not change at the end of the reaction. Therefore, powder samples could not be collected for XRD analysis.
2. The reference sample used for calculating the crystallinity index is a laser-synthesized saturation sample, which was prepared under synthesis conditions of 5 W power, 200 kHz repetition rate, and a synthesis duration of 300 minutes. The crystallinity values are based on individual experimental results.

**Table S2.** The crystallinity index (%) and yield (wt.%) values, and average particle sizes of laser-synthesized TPA-Silicalite-1 zeolites using different molar formulas.

|  | **M1^a)^** | | **M2^b)^** | | **M3^c)^** | |
| --- | --- | --- | --- | --- | --- | --- |
|  | **Laser** | **Hydro.** | **Laser** | **Hydro.** | **Laser** | **Hydro.** |
| ***C* [%]^d),e)^** | 91 | 100 | 89 | 100 | 81 | 100 |
| ***t* [h]** | 3 | 48 | 3 | 30 | 3 | 30 |
| ***Y* [wt.%]** | 69.7 | 69.2 | 55.6 | 59.9 | 41.7 | 56.6 |
| ***d_Avg_* ± SD [nm]** | 323.6 ± 35.2 | 203.8 ± 50.4 | 175.8 ± 17.5 | 102.7 ± 19.1 | 167.1 ± 16.9 | 99.5 ± 16.0 |
| ***CV* [%]** | 10.9 | 24.7 | 10 | 18.6 | 10.1 | 16.1 |

*C* – crystallinity, *t* - time, *Y* - yield, *d_Avg_* - average particle size and *CV* - coefficient of variation.

1. M1 = 25 SiO_2_: 9 TPAOH: 1450 H_2_O: 100 EtOH.
2. M2 = 25 SiO_2_: 9 TPAOH: 480 H_2_O: 100 EtOH.
3. M3 = 25 SiO_2_: 9 TPAOH: 450 H_2_O: 100 EtOH.
4. The reference samples for calculating the crystallinity index are three different hydrothermal-synthesized saturation samples prepared using three different molar formulas to make each comparison consistent. The crystallinity values are based on individual experimental results.
5. The average particle sizes of the crystals were determined from SEM images using Image J software. SD indicates standard deviation. The sample size (N) for particle size distribution analyses was 298.

**Table S3.** The crystallinity index (%) and yield (wt.%) values of laser-synthesized TPA-Silicalite-1 zeolites for different reaction times.

| **Molar Formula** | **t  [min]** | **Color** | **Y**  **[wt.%]** | **C**  **[%]^a)^** |
| --- | --- | --- | --- | --- |
| M1 | 70 | Light Milky | 9.3 | 48 |
|  | 90 | Milky | 11.6 | 55 |
|  | 120 | Opaque white | 46.5 | 62 |
|  | 150 | Opaque white | 60.4 | 69 |
|  | 180 | Opaque white | 69.7 | 78 |
|  | 200 | Opaque white | 69.7 | 88 |
|  | 220 | Opaque white | 74.4 | 96 |
|  | 240 | Opaque white | 72.0 | 99 |
|  | 260 | Opaque white | 74.4 | 98 |
|  | 280 | Opaque white | 72.0 | 99 |
|  | 300 | Opaque white | 76.7 | 100 |

*t* - time, *Y* - yield, and *C* - crystallinity.

1. The reference sample used for calculating the crystallinity index is a laser-synthesized saturation sample, which was prepared under synthesis conditions of 5 W power, 200 kHz repetition rate, and a synthesis duration of 300 minutes. The crystallinity values are averaged across five independent experiments.
